# Divergent roles for the RH5 complex components, CyRPA and RIPR in human-infective malaria parasites

**DOI:** 10.1371/journal.ppat.1007809

**Published:** 2019-06-11

**Authors:** Ellen Knuepfer, Katherine E. Wright, Surendra Kumar Prajapati, Thomas A. Rawlinson, Franziska Mohring, Marion Koch, Oliver R. Lyth, Steven A. Howell, Elizabeth Villasis, Ambrosius P. Snijders, Robert W. Moon, Simon J. Draper, Anna Rosanas-Urgell, Matthew K. Higgins, Jake Baum, Anthony A. Holder

**Affiliations:** 1 Malaria Parasitology Laboratory, The Francis Crick Institute, London, United Kingdom; 2 Department of Life Sciences, Imperial College London, London, United Kingdom; 3 Department of Biochemistry, University of Oxford, Oxford, United Kingdom; 4 Department of Biomedical Sciences, Institute of Tropical Medicine, Antwerp, Belgium; 5 The Jenner Institute, University of Oxford, Oxford, United Kingdom; 6 Department of Infection Biology, London School of Hygiene and Tropical Medicine, London, United Kingdom; 7 Proteomics Science and Technology Platform, The Francis Crick Institute, London, United Kingdom; 8 Departamento de Ciencias Celulares y Moleculares, Universidad Peruana Cayetano Heredia, Lima, Peru; University of Geneva, SWITZERLAND

## Abstract

Malaria is caused by *Plasmodium* parasites, which invade and replicate in erythrocytes. For *Plasmodium falciparum*, the major cause of severe malaria in humans, a heterotrimeric complex comprised of the secreted parasite proteins, PfCyRPA, PfRIPR and PfRH5 is essential for erythrocyte invasion, mediated by the interaction between PfRH5 and erythrocyte receptor basigin (BSG). However, whilst CyRPA and RIPR are present in most *Plasmodium* species, RH5 is found only in the small *Laverania* subgenus. Existence of a complex analogous to PfRH5-PfCyRPA-PfRIPR targeting BSG, and involvement of CyRPA and RIPR in invasion, however, has not been addressed in non-*Laverania* parasites. Here, we establish that unlike *P*. *falciparum*, *P*. *knowlesi* and *P*. *vivax* do not universally require BSG as a host cell invasion receptor. Although we show that both PkCyRPA and PkRIPR are essential for successful invasion of erythrocytes by *P*. *knowlesi* parasites *in vitro*, neither protein forms a complex with each other or with an RH5-like molecule. Instead, PkRIPR is part of a different trimeric protein complex whereas PkCyRPA appears to function without other parasite binding partners. It therefore appears that in the absence of RH5, outside of the *Laverania* subgenus, RIPR and CyRPA have different, independent functions crucial for parasite survival.

## Author summary

Malaria is one of the most devastating infectious diseases, causing significant human suffering and death. It is caused by parasites of the genus *Plasmodium* proliferating in the bloodstream. Understanding the mechanism of erythrocyte invasion is key for developing novel intervention strategies. *P*. *falciparum*, the cause of the most severe form of malaria, requires the interaction of a trimeric protein complex RH5-CyRPA-RIPR with the host receptor BSG for successful invasion. We show here that the BSG receptor is not essential for invasion by two other major causes of human malaria, *P*. *vivax* and *P*. *knowlesi*. Furthermore, we analyzed the role of CyRPA and RIPR in the absence of an RH5-like molecule in *P*. *knowlesi* and show that these molecules do not associate to form a protein complex unlike in the presence of RH5 in *P*. *falciparum*. PkRIPR is part of a different protein complex. Despite this difference CyRPA and RIPR still have essential functions during host cell invasion in other important human malaria-causing parasites.

## Introduction

Malaria, caused by *Plasmodium* parasites, remains a major world health problem with 219 million annual malaria cases and 435,000 deaths reported in 2017 [1]. While *P*. *falciparum* is responsible for most deaths, predominantly in sub-Saharan Africa, *P*. *vivax* is responsible for 34% of all cases outside of Africa and is the dominant cause of malaria in the Americas [2]. *P*. *knowlesi* is primarily a macaque parasite, but zoonotic infection can cause death and severe disease in humans, with a significant number of cases in Southeast Asia [3].

As part of their complex life cycle, *Plasmodium* parasites infect and replicate within erythrocytes to cause the clinical symptoms of malaria. This multi-step invasion process by *Plasmodium* merozoites is crucial for survival and transmission of the parasite. Significant advances have been made in dissecting this process, which typically takes less than 1 min [4]. This process involves the merozoite surface, apical microneme and rhoptry organelle secretion, and an actin-myosin parasite motor. Initial weak surface interactions between the merozoite and erythrocyte [5] precede strong deformations of the erythrocyte caused by erythrocyte binding-like (EBL) and reticulocyte binding-like (RBL) ligands released from apical organelles (the micronemes and rhoptry neck, respectively) onto the merozoite surface, binding to their specific host cell receptors. These interactions appear to mediate the merozoite reorientation, which is followed by the release of the rhoptries and moving junction formation between the parasite and the erythrocyte. As the merozoite invades, the junction likely acts as the bridge between the actin-myosin parasite motor and the host cell [6]. The invasion process is completed with the sealing of the parasitophorous vacuole (PV) and erythrocyte plasma membrane.

*Plasmodium* has evolved an extended repertoire of EBL and RBL proteins that allow for molecular redundancy in host cell recognition and invasion, likely a strategy to circumvent erythrocyte receptor polymorphisms and host immune responses (reviewed in [7]). Non-redundant exceptions are *P*. *falciparum* reticulocyte-binding protein homologue (RH) 5 [6], *P*. *vivax* Duffy binding protein (DBP) [8], and *P*. *knowlesi* DBPα, all of which are considered essential for human erythrocyte invasion [9,10].

PfRH5 is a highly conserved protein [11] that binds to BSG on the erythrocyte. Antibodies targeting either PfRH5 or BSG potently block invasion, as does the lack of BSG on an erythroleukemic BSG knockout cell line [12–16]. Its essentiality, its high degree of sequence conservation and its susceptibility to antibody targeting render PfRH5 a promising vaccine candidate, which has been confirmed experimentally using PfRH5-based formulations to protect *Aotus* monkeys against a virulent, heterologous *P*. *falciparum* challenge [17].

PfRH5 is localized to the rhoptries [6,18] and, upon secretion, forms a heterotrimeric complex with two micronemal proteins, cysteine rich protective antigen (PfCyRPA) and RH5-interacting protein (PfRIPR) [19,20]. This complex follows the moving junction during invasion [6,21]. Both PfCyRPA and PfRIPR are essential for invasion, and antibodies against either have shown invasion-blocking potential [19–21]. None of the trimeric complex components has a membrane anchor, but recently the GPI-anchored protein P113 has been proposed to anchor the complex to the merozoite surface [22].

Inhibiting the PfRH5-BSG interaction, or using inducible PfRIPR and PfCyRPA knockout parasites, indicates the complex functions during invasion following the strong deformations caused by EBL and RBL protein binding, and prior to moving junction formation [4,12,21]. The use of erythrocytes loaded with a Ca^2+^ reporter suggests that the PfRH5-BSG interaction may establish a physical connection between the cells, enabling Ca^2+^ diffusion from the merozoite into the erythrocyte [4,12,21,23]. Although the Ca^2+^ signal was only detected in ~50% of invasion events, a role was proposed for PfRH5-BSG in triggering rhoptry release, an essential step of invasion. Recently, multimeric RIPR-RH5 structures that form on and integrate into the erythrocyte membrane during invasion, were proposed [24]. It was hypothesized that these form pores, although no evidence for Ca^2+^ diffusion through these structures was provided.

Despite this apparent key role of RH5 in *P*. *falciparum* erythrocyte invasion, no orthologue of *Pfrh5* has been identified in the genomes of any other *Plasmodium* species beyond those closely related to *P*. *falciparum*, in the *Laverania* subgenus [11,25,26]. In contrast, orthologues of *Pfripr* and *Pfcyrpa* are found in all or most *Plasmodium* species, respectively. Therefore, reasoning that the PfRH5-RIPR-CyRPA complex must mediate a universally essential step during host cell invasion, we sought to determine whether PkRIPR and PkCyRPA are required for *P*. *knowlesi* growth in the absence of a PfRH5 orthologue, and whether these proteins are part of a protein complex involved in erythrocyte invasion by *P*. *knowlesi*. We further investigate what role BSG plays during erythrocyte invasion of two important human malaria-causing parasites, *P*. *knowlesi* and *P*. *vivax*. We show that unlike *P*. *falciparum* neither *P*. *vivax* nor *P*. *knowlesi* are wholly dependent on erythrocyte BSG for successful invasion. Using CRISPR/Cas9 gene editing paired with DiCre recombinase methodology to generated inducible gene deletions, we demonstrate here that both *Pkcyrpa* and *Pkripr* are essential for *P*. *knowlesi* survival *in vitro* and are involved in the erythrocyte invasion process. However, these proteins do not form a complex with each other; instead, PkRIPR is part of a different trimeric protein complex, whilst no binding partners were identified for PkCyRPA. We conclude that the essentiality of the PfRH5-RIPR-CyRPA complex binding to erythrocyte BSG is unique to *P*. *falciparum* and the *Laverania*, with divergent ligand-receptor complexes likely fulfilling this key mechanistic role in other *Plasmodium* species for successful erythrocyte invasion.

## Results

### Variable dependence on BSG for human erythrocyte invasion by three human malaria parasite species

**Fig 1:**
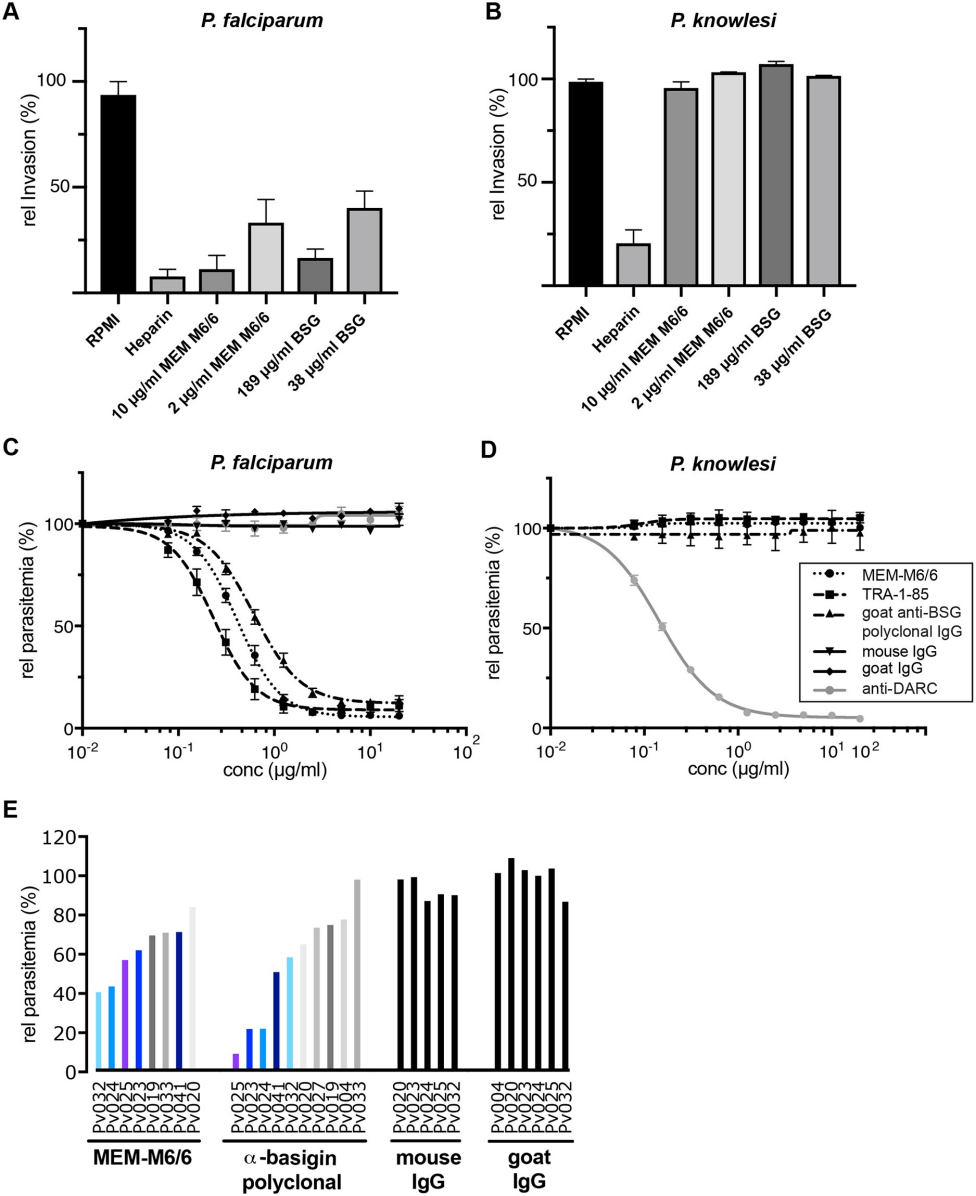
Role of basigin (BSG) as a receptor for erythrocyte invasion by *P*. *falciparum*, *P*. *knowlesi* and *P*. *vivax*. (**A**) Merozoite invasion inhibition assay in the presence of anti-BSG mAb MEM-M6/6 or BSG protein for *P*. *falciparum* and (**B**) *P*. *knowlesi*. Late schizonts of *P*. *falciparum* and *P*. *knowlesi* were manually ruptured and the merozoites added to fresh erythrocytes, either with no additives, with heparin, MEM-M6/6, or BSG. Invasion was quantified after 12 h using flow cytometry. Three independent experiments were performed for *P*. *falciparum* and two for *P*. *knowlesi* in triplicate. Invasion is normalised to the RPMI medium alone control, and mean and standard deviation are displayed. (**C**) Growth inhibition assay (GIA) using schizont cultures of *P*. *falciparum* and (**D**) *P*. *knowlesi* in the presence of serial dilutions of anti-BSG mouse mAbs MEM-M6/6 and TRA-1-85, anti-BSG goat polyclonal IgG, anti-DARC CA111 nanobody as well as non-specific mouse and goat IgG (legend in box on the right of plot D). Parasitemias of three independent experiments in triplicate were measured 40 h after setup using flow cytometry. Mean and standard error are displayed. (**E**) Invasion inhibition assay using anti-BSG antibodies on field isolates of *P*. *vivax*. Blood samples were taken from patients in Peru, matured to schizont stage *in vitro* and incubated with anti-BSG mAb MEM-M6/6 and anti-BSG goat polyclonal IgG, as well as non-specific mouse IgG and goat IgG at 10 μg/ml. Ring stage parasitemia was determined by microscopy of Giemsa-stained blood smears. The relative parasitemia (in %) is displayed for each individual sample compared to control treatment of the same sample. Color-coding highlights the same patient samples treated with either of the anti-BSG antibodies.

It has been demonstrated that BSG is an essential receptor for erythrocyte invasion by all tested *P*. *falciparum* strains, by blocking its binding function with either antibodies or soluble BSG [13,27]. Here, we investigated the importance of BSG as a receptor for erythrocyte invasion by *P*. *knowlesi* and *P*. *vivax*. Assays using *P*. *knowlesi* A1-H.1 merozoites [28] showed inhibition of invasion by heparin, but no inhibition by either an anti-BSG mouse mAb (MEM-M6/6) or soluble BSG at concentrations that severely inhibit *P*. *falciparum* invasion (Fig 1A and 1B). In growth inhibition assays (GIAs) over 48 h, increasing concentrations of three different anti-BSG antibodies (MEM-M6/6, TRA-1-85, and goat anti-BSG polyclonal IgG) all failed to inhibit *P*. *knowlesi* invasion and growth (Fig 1D). Nanobodies against the Duffy antigen receptor for chemokines (DARC), CA111 [29], inhibited growth severely with a half-maximum inhibitory concentration (IC_50_) of 0.15 μg/ml. The reliance of *P*. *knowlesi* on DARC as an invasion receptor has been well documented [10,30]. In parallel, the growth of *P*. *falciparum* (3D7) was severely inhibited in a dose-dependent manner by all three anti-BSG antibodies (Fig 1C) with IC_50_ values of 0.41 μg/ml (MEM-M6/6), 0.23 μg/ml (TRA-1-85) and 0.61 μg/ml (goat anti-BSG polyclonal IgG). The anti-DARC nanobody was not inhibitory, and neither were non-specific mouse and goat IgG.

We next investigated the role of BSG in invasion by *P*. *vivax*. Ten primary human isolates of *P*. *vivax* were tested in an invasion inhibition assay with 10 μg/ml of anti-BSG mAb MEM-M6/6, or goat anti-BSG polyclonal IgG (Fig 1E). Non-specific mouse and goat IgG at the same concentration were used as negative controls. Whereas non-specific IgGs had no effect on growth rates, both anti-BSG antibodies inhibited invasion of most isolates to varying degrees. Some isolates were inhibited by >90% (Pv025) whereas others showed little or no inhibition (Pv033). The differences in median parasitemia (S1 Fig) between control and anti-BSG antibody treatments were significant (p<0.01). However, the variable inhibition profile across different isolates suggests that *P*. *vivax* is not reliant on BSG as a host receptor, but rather may use BSG as one of several alternative receptors, which can be used by different parasite isolates to invade erythrocytes, a concept well known from *P*. *falciparum* [31–33]. As such, these data demonstrate that unlike *P*. *falciparum*, neither *P*. *knowlesi* nor *P*. *vivax* is reliant on BSG as a receptor for human erythrocyte invasion, suggesting that the absolute requirement for BSG engagement evolved in the *Laverania* subgenus with the emergence of RH5.

### RIPR and CyRPA are essential for *P*. *knowlesi* growth

RIPR and CyRPA are conserved across all or most *Plasmodium* species, (S2 Fig) with *cyrpa* absent from *P*. *berghei* and other rodent-infective *Plasmodium* parasites. Comparing mRNA expression profiles of *ripr* and *cyrpa* across species (RNAseq data from [34]) showed that both genes, and *rh5* (in *P*. *falciparum* only), are transcribed during blood-stage schizogony in *P*. *falciparum*, *P*. *vivax* and *P*. *knowlesi* (S3 Fig).

**Fig 2:**
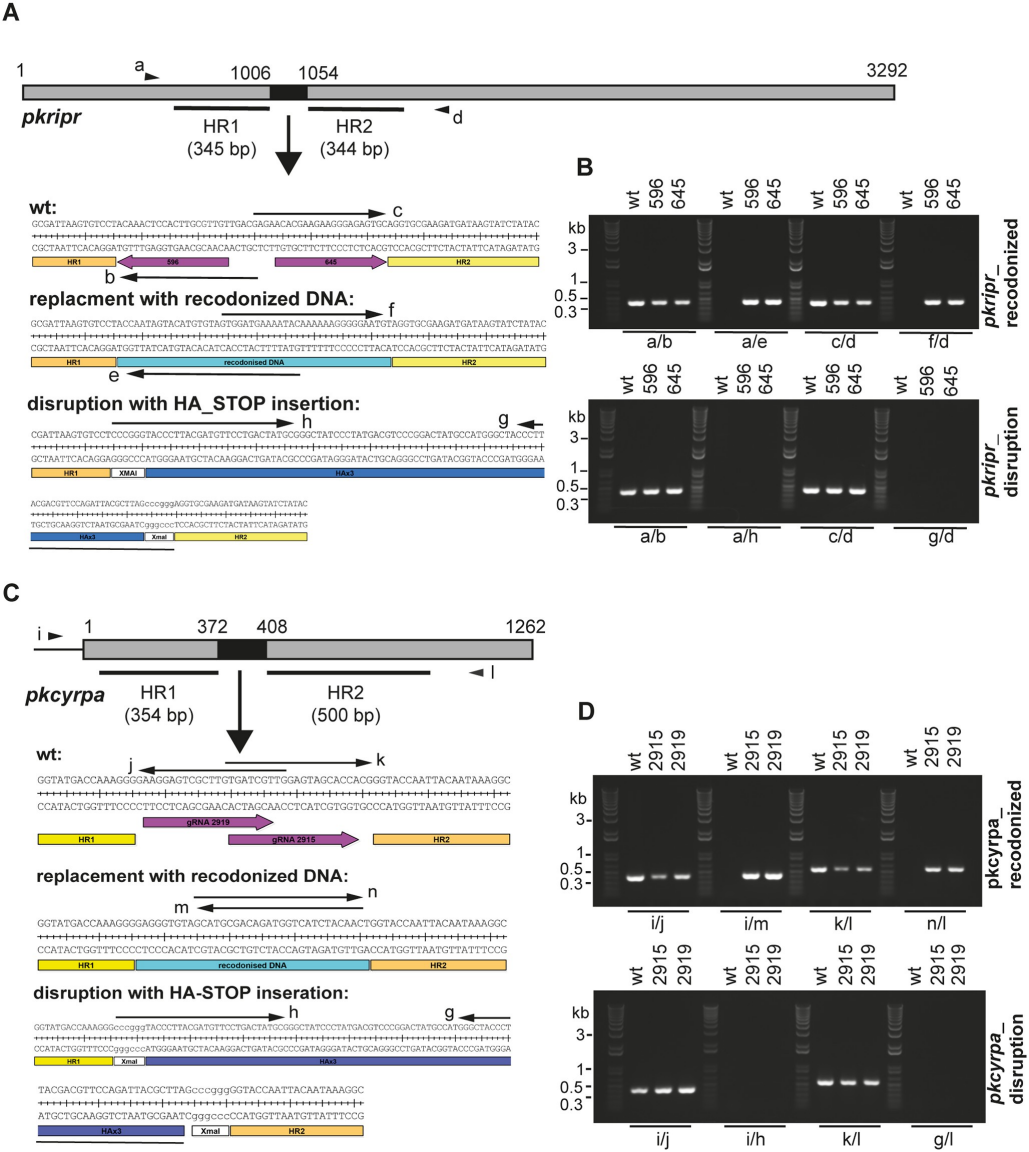
RIPR and CyRPA are essential for *P*. *knowlesi* parasite growth. Schematics of disruption approaches for *ripr* (**A**) and *cyrpa* (**C**) in *P*. *knowlesi*, respectively. ORFs are depicted, indicating regions used as homology regions (HR) in repair plasmids. Black boxes represent endogenous DNA sequence (bp 1006–1054 (*ripr*) and 372–408 (*cyrpa*)) which was replaced either by recodonized DNA or by a triple HA-tag followed by a stop codon. Sequence details are given in the corresponding boxes below, of endogenous (wild type), recodonized DNA or HA-STOP regions. Primers used in PCR to genotype transfectants are indicated by arrows. **B**) Genotyping of PkA1-H.1 transfections with pGEMT-PkRIPRko or pGEMT-PkRIPRrecodonized with pDC596 and pDC645 Cas9/guide plasmids. **D**) Genotyping of PkA1-H.1 transfections using pGEMT-PkCyRPAko or pGEMT-PkCyRPArecodonized together with Cas9/guide plasmids pDC2915 or pDC2919. Primers for genotyping (S2 Table) were as follows: a, PKRIPRkoextFor; b, PKRIPRintRev; c, PKRIPRintFor; d, PKRIPRkoextRev; e, PKRIPRkorecRev; f, PKRIPRkorecodFor; g, HArev(Xma); h, HAfor(Xma); i, PKCyRPAextFor; j, PKCyRPAintRev; k, PKCyRPAintFor; l, PKCyRPAextRev; m, PKCyRPArecodRev; n, PKCyRPArecodFor. Expected PCR product sizes for *Pkripr* are: a/b = 426 bp; a/e = 434 bp; c/d = 412 bp; f/d = 418 bp; a/h = 509 bp; g/d = 499 bp. Expected PCR product sizes for *Pkcyrpa* are: i/j = 425 bp; i/m = 438 bp; k/l = 566 bp; n/l = 570 bp; i/h = 504 bp; g/l = 654 bp.

The function of CyRPA and RIPR in the life cycle of *Plasmodium spp*. other than *P*. *falciparum* is undefined. Given the importance of PfRIPR and PfCyRPA, possibly in rhoptry release and Ca^2+^ signaling in the host cell, we sought to determine the effect of reducing or abolishing expression of their *P*. *knowlesi* orthologues. We investigated whether disruption of *ripr* and *cyrpa* in *P*. *knowlesi* is feasible. Using a newly developed CRISPR/Cas9 gene modification vector, we attempted to disrupt *ripr* and *cyrpa* by insertion of a triple-HA epitope tag followed by a stop codon in each ORF, or as control, replacing the same region with recodonized DNA sequence (Fig 2A and 2C). A 48 bp sequence of *ripr* was successfully replaced by recodonized sequence in duplicate experiments using two independent guides (Fig 2B, primer pairs a/e and f/d) whereas integration of the epitope tag with stop codon was unsuccessful (Fig 2B, primer pairs a/h and g/d). Similarly, replacement of a 36 bp region of *cyrpa* with recodonized sequence was successful in duplicate experiments using two guides (Fig 2D, primer pairs i/m and n/l) but not replacement with a reporter-stop sequence (Fig 2D, primer pairs i/h and g/l). This strongly implies that *Pkripr* and *Pkcyrpa* disruption leads to non-viable parasites.

### RIPR forms a novel CyRPA-independent complex in *P*. *knowlesi*

**Fig 3:**
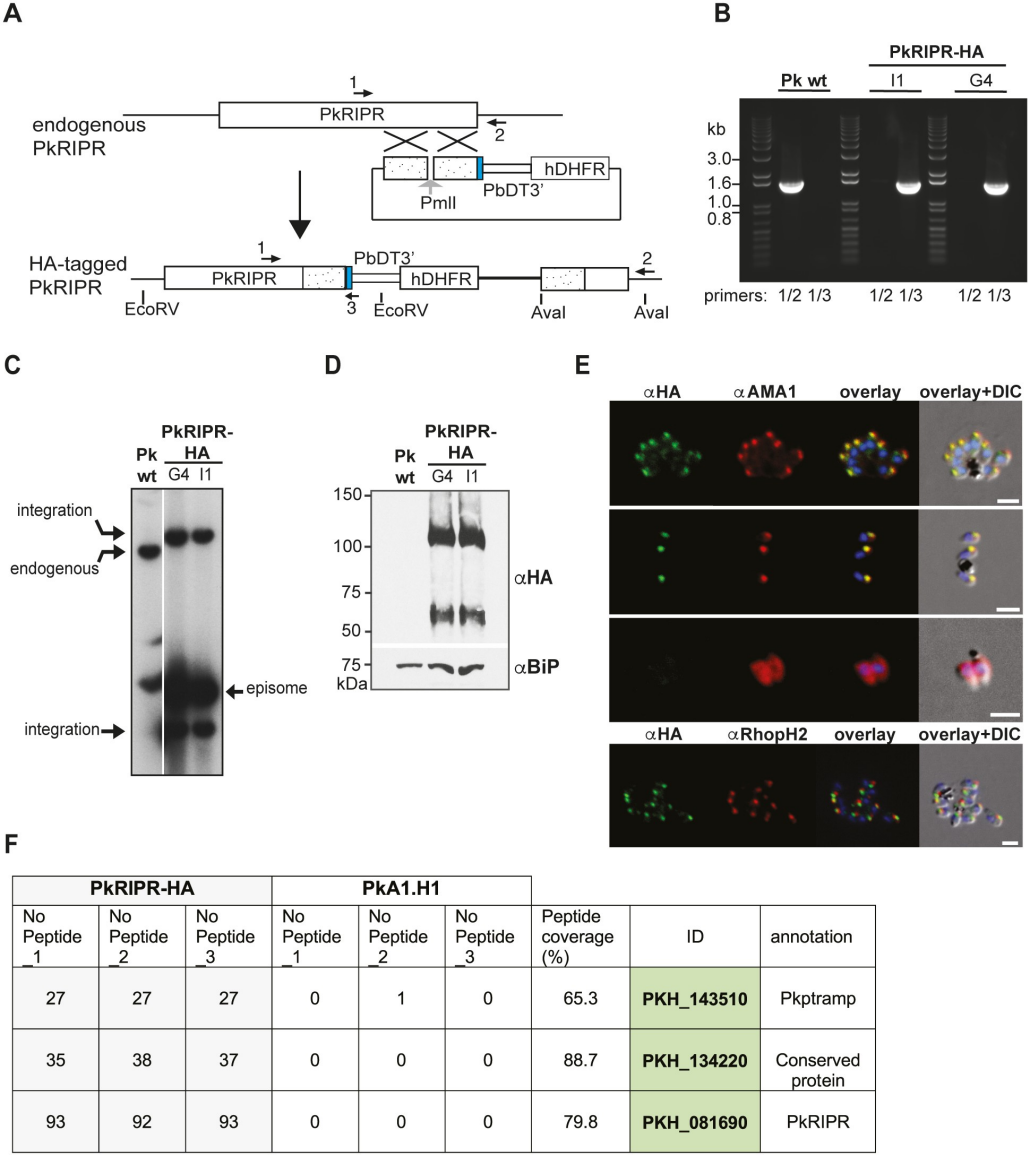
HA-tagging, localization and complex formation of PkRIPR. (**A**) Schematic of single homologous recombination to generate C-terminally tagged parasite line, PkRIPR-HA. (**B**) Integration PCRs using genomic DNA from PkA1-H.1 (PK wt) parasites or PkRIPR-HA parasite clones I1 and G4. Using the primer pair PkRIPRextF1/PkRIPRutrRev, a 1595 bp fragment was amplified from wild type parasites, whereas primer pair PkRIPRextF1/HArev amplified a 1526 bp fragment only from transgenic PkRIPR-HA parasites. Positions of primer pairs are indicated in schematic (A) and size standards (kb) on the left of the gel (B). Primers used were PkRIPRextF1 (1), PkRIPRutrRev (2), HArev (3). (**C**) Southern blot of wildtype and transgenic parasite DNA digested with EcoRV/AvaI. Endogenous locus band, integration and episomal bands are indicated with arrows. (**D**) Immunoblot of purified schizont material solubilized in Laemmli sample buffer probed with anti-BiP and anti-HA (3F10) antibodies. Molecular mass standards are indicated on the left in kDa. (**E**) IFAs of schizonts and merozoites with micronemes not secreted (2^nd^ row) or secreted (3^rd^ row) were probed with anti-HA antibody (green), anti-PkAMA1 antibody, or anti-PkRhopH2 antibody (both red) and DAPI (Blue). The third panel shows an overlay of DAPI marking the nucleus with anti-HA and anti-PkAMA1; the fourth panel includes a differential interference contrast (DIC) image of the whole parasite. Scale bar = 2 μm. (**F**) Table of proteins interacting with PkRIPR-HA as identified by LC-MS/MS following immunoprecipitation. The three top-scoring, consistently identified proteins are listed. The number of peptides of three technical replicates of immunoprecipitates from PkRIPR-HA clone G4 or wild type parasites is displayed, as is the total peptide coverage, gene IDs and annotations (PlasmoDB release 25).

To explore whether PkCyRPA and PkRIPR form a complex in the absence of RH5 in *P*. *knowlesi*, we generated a transgenic PkRIPR-HA-tagged *P*. *knowlesi* line (Fig 3A). Successful integration was confirmed by PCR (Fig 3B) and Southern Blot analyses (Fig 3C). Immunoblotting of purified late schizont stages, probed with an anti-HA antibody (3F10), revealed the expected ~120 kDa PkRIPR-HA band, and a ~60 kDa band (Fig 3D). The presence of the smaller band suggests PkRIPR is processed like PfRIPR [19], probably between the two clusters of EGF domains (S2 Fig).

In *P*. *falciparum*, PfRIPR is present in the micronemes and following secretion forms the trimeric complex with PfRH5 and PfCyRPA [19,20]. Indirect immunofluorescence assays (IFAs) using anti-HA antibodies and the PkRIPR-HA parasite clone G4 showed an apical localization for PkRIPR-HA in late-stage schizonts (Fig 3E). Co-localization experiments with antibodies to PkAMA1, a micronemal protein, confirmed that PkRIPR-HA is likely micronemal. Following micronemal secretion, PkAMA1, a type 1 transmembrane protein, is present on the merozoite surface, while the immunofluorescence signal for PkRIPR-HA is lost (Fig 3E); PkRIPR, like PfRIPR, lacks any membrane-anchoring domain. These data suggest that PkRIPR and PfRIPR have a conserved localization in the micronemes. PfRIPR has been shown to be released onto the merozoite surface at invasion and follows the moving junction as the merozoite invades [19,21]. The signal of PkRIPR is lost from purified merozoites after microneme secretion (visualized by the fluorescence signal of anti-AMA1 changing from apical dots to a circle outlining the merozoite surface).

To determine whether PkCyRPA and PkRIPR interact in *P*. *knowlesi*, we conducted co-immunoprecipitation experiments using two different anti-HA matrices to purify PkRIPR-HA and interacting proteins, following the same approach used with *P*. *falciparum* [19]. Immunoprecipitations from extracts of purified schizonts or concentrated culture supernatant from PkRIPR-HA parasites gave comparable results. Two proteins were consistently identified by LC-MS/MS in the co-precipitate with PkRIPR-HA (Fig 3F, S1 Dataset); however, PkCyRPA was not found. The two identified proteins have a predicted signal peptide, and their gene transcription profiles are similar to that of PkRIPR, upregulated late in schizogony. These proteins are *P*. *knowlesi* PTRAMP (PKH_143510) and a hypothetical protein (PKH_134220) that is conserved within the genus and which we term cysteine-rich, small, secreted protein (CSS) here. No information is available about orthologues of *Pkcss* except that *css* has an essential role in the asexual blood stage of *P*. *falciparum* [35]. PTRAMP, a member of the thrombospondin-related anonymous protein (TRAP) family, is located apically in *P*. *falciparum* merozoites [36], and is essential for *P*. *berghei* and *P*. *falciparum* blood-stage development [35,37,38]. PfPTRAMP is a type 1 membrane protein that is transferred to the merozoite surface where it forms a dimer. The subtilisin-like protease PfSUB2 cleaves PfPTRAMP from the merozoite surface during invasion releasing the protein into the culture supernatant [39]. Analysing peptide sequences identified by LC-MS/MS from these co-immunoprecipitations revealed that PkPTRAMP peptides ELYENVAGR and GAEKELYENVAGR derived from the cytoplasmic C-terminus were only detected in PkRIPR-HA precipitates from schizont lysates, not in precipitates from culture supernatant. In contrast, peptide ETRPCQVPLPPCNSLFELK derived from the extracellular domain just N-terminal to the predicted transmembrane domain of PkPTRAMP was detected in both. This demonstrates that, in the absence of RH5, RIPR is associated with an entirely different, trimeric protein complex in *P*. *knowlesi*, lacking CyRPA and any RH5-like protein, with PkPTRAMP likely anchoring this new trimeric complex to the merozoite surface.

### The PkRIPR trimeric protein complex and PkCyRPA are essential for successful erythrocyte invasion

To investigate in more detail when and where during the parasite’s asexual blood stage the newly identified PkRIPR-PkPTRAMP-PkCSS complex as well as PkCyRPA function, we generated an inducible gene knockout (iKO) system for *P*. *knowlesi* based on the DiCre recombinase approach applied so successfully in *P*. *falciparum* [40]. For this we modified the pSKIP vector [41] and targeted insertion of the DiCre cassette into the *pfs47* homologue of *P*. *knowlesi*, PKNH_1254100, using CRISPR-Cas9 guide plasmid pDC946. This resulted in the DiCre-recombinase expressing cloned parasite line PkpSKIP9-10. Next we floxed endogenous genes *ptramp* and *css* by inserting a *loxPint* cassette (see Materials and Methods for detail) downstream of the predicted signal peptide sequence and a *loxP* cassette following the stop codon (S4 Fig). At the same time we inserted a triple HA-tag sequence between the predicted signal peptide and the *loxPint* sequence. Transfectants were cloned by limiting dilution and clones analyzed by PCR (S4 Fig). PCR products of inducible Pkptramp parasite clones were of expected sizes (733 bp for primer pair 4/7 and 506 bp for primer pair 5/9) and no bands were detected with primers amplifying wild type sequence (primer pair 4/6 expected wild type band of 723 bp; primer pair 5/8 expected wild type band of 804 bp). Similarly, PCR product sizes of inducible Pkcss parasites were as expected with primer pair 10/14 amplifying a 783 bp band, and primer pair 11/15 a 613 bp band. Again, no bands corresponding to amplification of wild type *css* sequence were detected (primer pair 10/12 expected wild type band of 484 bp; primer pair 11/13 expected wild type band of 729 bp). Inducible knockout parasites for *cyrpa* were generated by replacing the endogenous intron with the *loxPint* cassette and placing a *loxP* sequence after the stop codon (S4 Fig). To detect PkCyRPA protein a triple HA-tag sequence was encoded located 5’ to the stop codon. After limiting dilution cloning of transfectants, parasites were analyzed by PCR, showing integration bands for primer pair 16/19 of 577 bp and, primer pair 17/21 of 904 bp (S4 Fig). No wild type DNA sequence for *cyrpa* could be detected in the transgenic clones, whereas genomic DNA (PkpSKIP9-10) gave amplification products of 641 bp with primer pair 16/18 and 670 bp with primer pair 17/20 as expected.

Having generated HA-tagged transgenic parasites we investigated the subcellular location of PkPTRAMP, PkCSS and PkCyRPA in these lines. IFAs using anti-HA antibody 3F10 together with anti-AMA1 or anti-RhopH2 antibodies showed partial co-localization with the micronemal marker (AMA1) but none with the rhoptry bulb marker RhopH2 (S5 Fig).

To confirm the formation of the PkRIPR-PkPTRAMP-PkCSS complex in schizonts of *P*. *knowlesi* parasites and the absence of PkCyRPA from this complex we immunoprecipitated HA-tagged parasite proteins from schizont lysates of PkptrampiKO, PKcssiKO and PKcyrpaiKO, using 3F10 affinity matrix. Analysis of all co-immunoprecipitated proteins by LC-MS/MS identified PkPTRAMP, PkRIPR and PkCSS as the three most abundant proteins from PkptrampiKO lysate (S2 Dataset). Equally, the same proteins were identified as most abundant in the immunoprecipitation from PkcssiKO lysates. However, co-immunoprecipitation experiments using the 3F10 matrix on PkcyrpaiKO schizont lysate only identified peptides derived from CyRPA abundantly enriched compared to the negative control (S2 Dataset).

**Fig 4:**
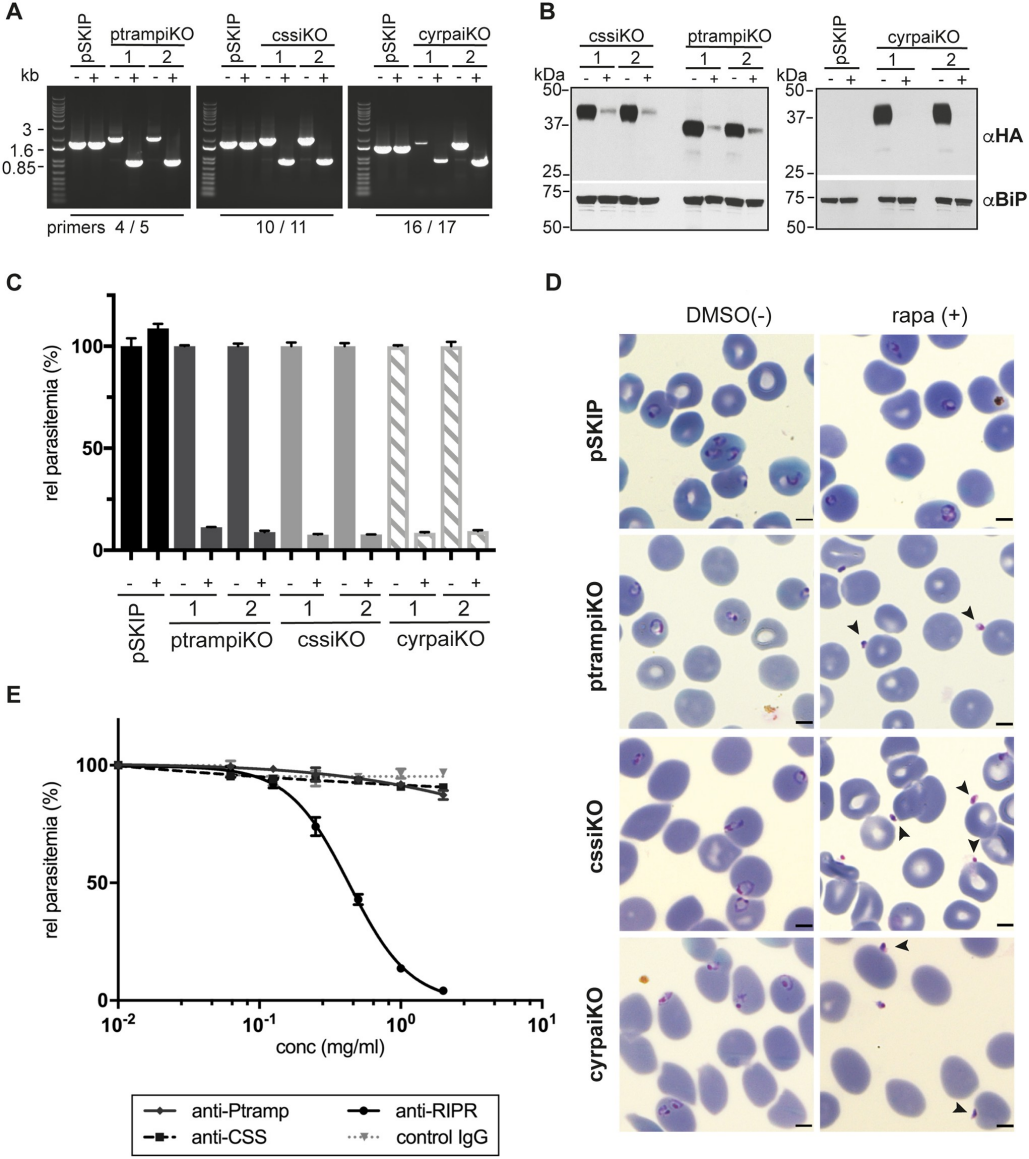
Newly identified complex components PkRIPR, PkCSS and PkPTRAMP are essential for human erythrocyte invasion, as is PkCyRPA. Inducible knockouts of *pkcss*, *pkptramp* and *pkcyrpa* were produced in the *P*. *knowlesi* DiCre expressing PkpSKIP 9–10 parasite line using CRISP/Cas9 gene editing. (**A**) PCR screen of wild type (pSKIP) and two clones of each transgenic parasite line (ptrampiKO, cssiKO, cyrpaiKO) showing successful and complete recombination of floxed gene sequences after rapamycin addition (+). Primer pair 4/5 specific for the *ptramp* locus amplified a 1741 bp band in wild type parasites, a 1993 bp band in mock -treated (-), transgenic parasites and a reduced size band of 965 bp after excision induced by rapamycin (+). Primer pair 10/11 specific for the *css* locus amplified a 1761 bp band in wild type, a 2013 bp band in mock-treated, transgenic parasites and a 919 bp band after excision. Primer pair 16/17 specific for the *cyrpa* locus amplified a 1467 bp band in wild type, a 1559 bp band in mock-treated and a 891 bp band in rapamycin-treated, transgenic cyrpaiKO parasites. DNA marker sizes are shown on the left. (**B**) Immunoblot of schizonts purified 26 h after DMSO (-) or rapamycin (+) treatment of ring stage cultures from wild type (pSKIP) and inducible knockout parasite lines. Two clones are shown for each knockout parasite. Blots were probed with anti-HA and anti-BiP antibodies as indicated on the right of the panels. All protein signals correspond approximately with their predicted molecular masses (HA-tagged PkCSS has a predicted molecular mass of 40.6 kDa, HA-tagged PkPTRAMP a predicted molecular mass of 39.2 kDa and PkCyRPA-HA a predicted mass of 42.5 kDa). Molecular mass standards are indicated on the left. (**C**) Invasion assay of wild type and inducible knockout parasites lines over one growth cycle. Parasitemias were measured by flow cytometry 40 h after rapamycin / DMSO treatment. Means and standard errors of three independent experiments carried out in triplicate are displayed. (**D**) Giemsa-stained brightfield images of wild type and inducible knockout parasites 30 h after DMSO (-) or rapamycin (+) treatment. Arrowheads point to merozoites attached to erythrocytes which have failed to invade. Scale bars equal 2 μm. (**E**) Growth inhibition assay of PkA1-H.1 parasites over one cycle with serial dilutions of purified IgG raised against recombinantly produced PkPTRAMP, PkCSS, PkRIPR or control rabbit IgG. Parasitemias were determined by flow cytometry 18 h after assay setup at schizont stage. Means and standard errors of three independent experiments in triplicate are displayed.

To study the phenotype of gene knockouts, synchronized ring stage parasites from two clones of each inducible knockout line (ptrampiKO, cssiKO, cyrpaiKO) as well as the DiCre recombinase-expressing *P*. *knowlesi* parasite line PkpSKIP9-10 were treated with DMSO or 10 nM rapamycin for 28 h. Recombination induced by the addition of rapamycin resulted in complete excision of the floxed DNA sequence (Fig 4A) and loss of the protein as detected by immunoblots labelled with anti-HA antibodies (Fig 4B). A significant reduction in protein level for PkCSS and PkPTRAMP and complete lack of PkCyRPA can be seen in immunoblots after rapamycin treatment. Also, these inducible knockout parasites failed to complete their asexual life cycle as shown by invasion assay (Fig 4C). Rapamycin-treated PkpSKIP9-10 parasites however showed no invasion defect. Giemsa-stained thin blood smears (Fig 4D) clearly demonstrate that merozoites of knockout parasites PkptrampiKO, PkcssiKO and PkcyrpaiKO bound to the surface of human erythrocytes but failed to invade, unlike the DMSO-treated transgenic lines or the rapamycin-treated PkpSKIP9-10 DiCre parasites.

We also addressed the role of PkRIPR, PkCSS and PkPTRAMP in host cell invasion by invasion inhibition assays using rabbit antibodies generated against recombinant PkRIPR, PkPTRAMP and PkCSS proteins (S6 Fig; Fig 4E). Whereas control, anti-PTRAMP, and anti-CSS IgG at concentrations ranging from 0–2 mg/ml showed no reduction in parasitemia levels, anti-RIPR IgG reduced parasitemia levels severely, with an IC_50_ value of 0.43 mg/ml. Together, these data demonstrate the essential role the PkRIPR-PkPTRAMP-PkCSS complex plays during host cell invasion.

### PkPTRAMP binds to human erythrocytes

**Fig 5:**
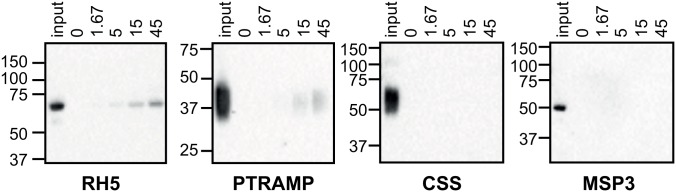
Erythrocyte binding assay demonstrating the ability of PTRAMP but not CSS to bind to receptors on the erythrocyte surface. Increasing amounts of recombinant proteins (in μg) were incubated with human erythrocytes, before repeated separation steps through oil and elution of bound protein. Immunoblots of eluted proteins are shown, probed with anti-His antibody. Recombinant RH5 and MSP3 were used as positive and negative controls respectively. Molecular mass standards are indicated on the left of each panel (in kDa).

PfPTRAMP has previously been shown to bind to a receptor on the surface of human erythrocytes [36]. We used recombinant PkPTRAMP and PkCSS to investigate their potential to bind to the surface of human erythrocytes (Fig 5). Recombinant His-tagged PfRH5 and PfMSP3 were used as positive and negative controls respectively. Immunoblots in Fig 5 show that binding to human erythrocytes was only detected with PkPTRAMP and RH5, but not PkCSS.

In summary, PkRIPR forms a complex with PkPTRAMP and PkCSS. This complex plays an essential role during erythrocyte invasion by *P*. *knowlesi* merozoites, as does PkCyRPA. All four proteins appear possibly to localize to micronemes, but in contrast to the situation in *P*. *falciparum* PkCyRPA and PkRIPR work independently in *P*. *knowlesi* and therefore have likely diverged in functional roles over the course of *Plasmodium* evolution.

## Discussion

In this work, we set out to characterize the role of CyRPA and RIPR in *P*. *knowlesi* that lacks an RH5 orthologue, and to investigate the use of BSG as a receptor for human erythrocyte invasion in two important human malaria-causing agents, *P*. *knowlesi* and *P*. *vivax*.

The RH5-BSG interaction is essential for invasion in all strains of *P*. *falciparum*. PfRH5 has been implicated in *P*. *falciparum* host cell tropism, with amino acid substitutions in both RH5 and BSG involved [11,42–44]. While the host cell specificity determinants for *P*. *vivax* and *P*. *knowlesi* are largely unknown [7,45,46], we show here that BSG is not an essential requirement for erythrocyte invasion in these species.

With *P*. *vivax* field isolates, anti-BSG antibodies gave varying levels of growth inhibition, and therefore BSG may be used as one of the alternative, redundant invasion receptors in *P*. *vivax*, similar to a recently identified reticulocyte-specific transferrin-receptor 1 [47] in *P*. *vivax* or glycophorins or CR1 in *P*. *falciparum* invasion [48–52]. PvTRAg5 (also known as PvTRAg38) has been shown to bind to BSG [53], perhaps serving as a ligand in an invasion pathway for some parasites.

Addressing the role of RIPR and CyRPA in the *P*. *knowlesi* asexual life cycle, we show that both proteins are essential to parasite survival but do not interact with each other, suggesting independent functions, unlike in *P*. *falciparum*. PkRIPR appears micronemal and interacts with two new binding partners, PkCSS and PkPTRAMP, which were reproducibly co-immunoprecipitated. Both proteins are conserved throughout the genus. CSS is a small cysteine-rich, secreted protein expressed during blood-stage schizogony, and its *P*. *falciparum* orthologue appears essential for parasite growth [35]. PTRAMP is an apical protein [38] essential in blood-stage development of *P*. *berghei* and *P*. *falciparum* [35,37]. In *P*. *falciparum* the protein is transferred to the merozoite surface as a membrane-bound dimer before cleavage by SUB2 to release a soluble product [39]. Further, PTRAMP has been reported to bind directly to erythrocytes, and anti-PTRAMP antibodies partially inhibit invasion at high antibody concentrations [36].

By developing an inducible gene regulation approach for *P*. *knowlesi* and by generating invasion inhibitory antibodies we could show that the PkRIPR-PkPTRAMP-PkCSS complex functions during invasion of host erythrocytes. The invasion assay using antibodies against all three complex components only showed inhibition of invasion with anti-RIPR antibodies. Anti-RIPR antibodies were raised to two small regions of PkRIPR homologous to regions in PfRIPR which previously showed potential as targets for growth inhibition of *P*. *falciparum* [19]. Antibodies against PkCSS and PkPTRAMP did not inhibit invasion. Given that we have shown that PkPTRAMP can bind to human erythrocytes, it was expected that specific antibodies would prevent this binding and be invasion inhibitory. It is possible that these proteins are not accessible to antibody during invasion, or that higher antibody concentrations are required, as seen with anti-PTRAMP antibodies in *P*. *falciparum* [36]. We have previously shown that DiCre recombinase-mediated gene excision after rapamycin addition takes >24 h to achieve ~80% excision levels in *P*. *falciparum* [40]. On immunoblots small amounts of PkCSS and PkPTRAMP are visible after rapamycin treatment (Fig 4B) although DNA excision appears complete. We presume that after rapamycin treatment the small number of ring stage parasites in the invasion assays (Fig 4C) reflects the residual protein level seen in the immunoblots and that during the ~28 h life cycle of *P*. *knowlesi* not all floxed genes in each parasite were excised prior to translation initiation.

For PkCyRPA, no binding partners were identified. However, like the PkRIPR-PkPTRAMP-PkCSS complex, PkCyRPA is important in host cell invasion, as shown here using the inducible gene knockout approach. Given that PkCyRPA acts independently of PkRIPR and that BSG is not a receptor for host cell entry by *P*. *knowlesi*, CyRPA has a different role during invasion in *P*. *falciparum* compared to *P*. *knowlesi*. This change of function during evolution is likely a consequence of the acquisition of both *rh5* and *cyrpa* by *P*. *falciparum* via horizontal gene transfer from a gorilla-infective *P*. *alderi* parasite [25,26]. This transfer resulted in a conserved synteny and presumably functionality of *cyrpa* and *rh5* only in *Laverania* species.

**Fig 6:**
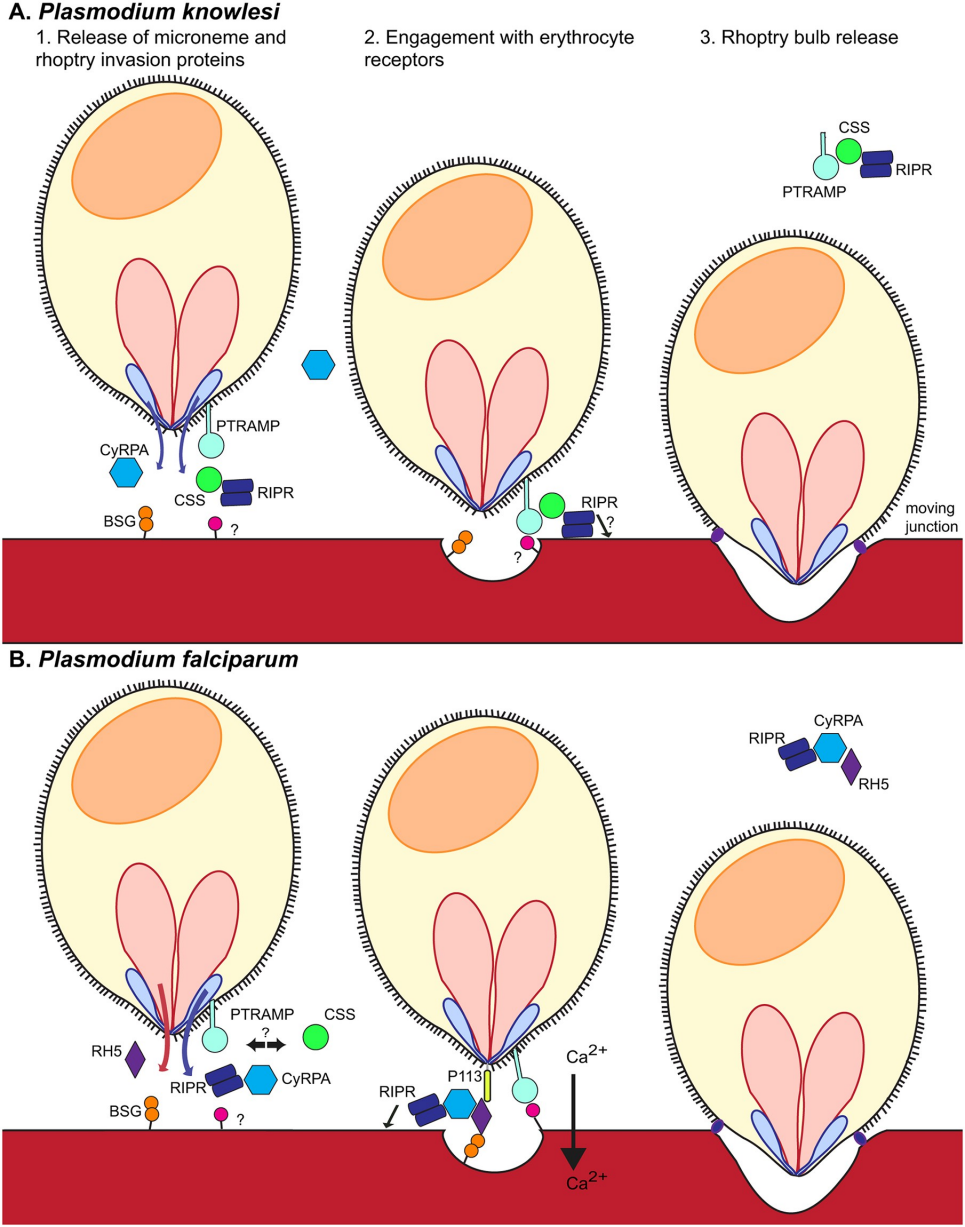
A cartoon depicting invasion of (A) *P*. *knowlesi* or (B) *P*. *falciparum* merozoites into human erythrocytes. *P*. *knowlesi* lacks an RH5-like molecule, resulting in the secretion of RIPR complexed with PkPTRAMP and PkCSS. The precise role of this complex in the invasion process is currently unknown but we speculate that PkPTRAMP as a transmembrane protein is both displaying PkRIPR and PkCSS on the merozoite surface after microneme release and is recognizing a receptor on the human erythrocyte, which is an important step required for successful invasion. BSG is not used as a human erythrocyte receptor by *P*. *knowlesi*. In stark contrast, *P*. *falciparum* RH5 and the micronemal proteins PfRIPR and PfCyRPA form a trimeric complex following rhoptry neck and microneme secretion. This complex localizes to the merozoite surface, through an interaction between the N-terminus of RH5 and GPI-anchored protein P113, and connects the parasite via BSG to the host erythrocyte. This interaction between RH5 and BSG is speculated to result in rhoptry bulb secretion and Ca^2+^ influx into the host cell. RH5 and RIPR have been postulated to multimerize and integrate into the erythrocyte membrane, which by an unknown mechanism results in the influx of Ca^2+^ into the host cell. This is followed by the formation of the moving junction. The RH5 complex follows the moving junction, from the apical tip of the merozoite to its posterior end, setting in motion the irreversible process of active invasion of the host cell by the merozoite.

Summarizing our data, we present a model of the different, essential roles of RIPR, CyRPA, and RH5 during erythrocyte invasion by *P*. *knowlesi* and *P*. *falciparum* (Fig 6A and 6B, respectively). In *P*. *knowlesi*, BSG engagement is not required during invasion of human erythrocytes. PkRIPR forms a complex with membrane-bound PkPTRAMP and a conserved, uncharacterized protein we named PkCSS (Fig 6A). This complex is involved in invasion, presumably mediating host cell recognition by interaction of PkPTRAMP with an unknown host cell receptor. PfPTRAMP is shed during invasion [39]. PkPTRAMP and PfPTRAMP are highly conserved, and peptides corresponding to the cytoplasmic C-terminus of PkPTRAMP were identified in immunoprecipitations from parasite schizont lysates, but not from culture supernatant, leading us to suggest that PkPTRAMP is similarly shed during invasion of *P*. *knowlesi* releasing the trimeric PkRIPR-PkPTRAMP-PkCSS complex from the merozoite surface. Whether or not PkRIPR integrates into the erythrocyte membrane as described for *P*. *falciparum* [24] is unclear. PkCyRPA, meanwhile, also localizes to micronemes and is involved in erythrocyte invasion, but no interaction partners have been identified for this protein. This is unlike the situation in *P*. *falciparum* where CyRPA forms a complex with RIPR and RH5 and BSG engagement is required during invasion of human erythrocytes. In *P*. *falciparum*, PfRH5 is present in the rhoptry neck, while RIPR and CyRPA are in micronemes, and following release early in invasion, the proteins form a trimeric complex that interacts with erythrocyte surface BSG (Fig 6B). Following binding to BSG, RIPR and RH5 form multimers which insert into the erythrocyte membrane [24]. By an unknown mechanism this results in Ca^2+^ influx into host erythrocyte, a process deemed important for invasion. PfPTRAMP and PfCSS appear to play no known role in the PfRH5-CyRPA-RIPR-BSG interaction, but might have a different, undiscovered role in invasion.

Ligand-receptor interactions involved in the initiation of invasion as well as the final step, moving junction formation, are conserved between *Plasmodia*. From our work, it appears that the presumed trigger for rhoptry secretion, a universally important invasion step, is species-specific, with the RH5-CyRPA-RIPR complex fulfilling this role only in *Laverania* parasites. With vaccine development in mind, understanding the critical steps and essential immunogens of host cell invasion across *Plasmodium* species is critical for the design of malaria intervention strategies.

## Materials and methods

### Ethical statement

Ethical approval for the collection of *P*. *vivax* isolates from infected patients in Peru was obtained from the Institutional Ethics Committees of the Universidad Peruana Cayetano Heredia, Lima, Peru. Ethical approval for the use of *P*. *vivax* isolates and collection of blood samples from hemochromatosis patients was obtained from the Ethics Committee of the Institute of Tropical Medicine (ITM), Antwerp, Belgium (946/14).

### Parasite culture

*P*. *falciparum* was maintained in human erythrocytes using standard conditions [54]. *P*. *knowlesi* was maintained using a modified complete RPMI 1640+AlbumaxII medium supplemented with 10% horse serum [55].

### Genetic modification of parasites and genotyping

The Pk*ripr* gene was modified by addition of sequence coding for a C-terminal epitope tag. For this, we used a modified PkconGFP vector [56,57], carrying a triple HA-tag. A 1331 bp region of PkRIPR was amplified using primers pHH4RIPRfor/rev and cloned into pHH4PkHA using XmaI/AvrII. The resulting plasmid was linearized using PmlI and transfected into PkA1-H.1 as described [57].

For genotyping, we employed diagnostic PCR, Southern blotting and immunoblotting approaches. For Southern blots, wild type and transgenic parasite genomic DNA was digested using EcoRV/AvaI. Fragments were separated by agarose gel electrophoresis and transferred to a nitrocellulose membrane before hybridization with a ^32^P-labelled PkRIPR-specific 1331 bp probe amplified using primer pair pHH4RIPRfor/rev. All primer sequences are listed in S2 Table. For immunoblotting, purified schizont and merozoite samples were solubilized in reducing sample buffer and proteins were resolved on NuPAGE Tris-Acetate or Bis-Tris gels before transfer to nitrocellulose membranes. Membranes were probed with anti-HA 3F10 mAb and anti-BiP rat antibodies, and detected using ECL.

The CRISPR-Cas9 vector for use in *P*. *knowlesi*, pDC2-PK-Cas9-hDHFRyFCU (S7 Fig) was generated by digestion of the *P*. *falciparum* CRISPR/Cas9 vector pDC2-Cas9-hDHFRyFCU [40] with NcoI/AvrII to replace the promoters driving *cas9* and the *hdhfr-yfcu* transcription with *P*. *knowlesi* specific promoters. For this, 5’UTR regions of Pk*hsp86* and Pk*ef1alpha* were amplified using primer pairs Pkhsp86promforNcoSpeI/Pkhsp86promRevAvrII and Pkef1aFor(SpeI)/Pkef1aRev(NcoI) from *P*. *knowlesi* genomic DNA before digestion and ligation into pDC2-Cas9-hDHFRyFCU. Finally, the U6 cassette was replaced with a synthetically generated DNA construct comprising 632 bp of 5’UTR of the *P*. *knowlesi* U6 spliceosomal RNA gene PKNH_1260300 and 100 bp of its terminator sequence flanking the tracrRNA and BbsI site for protospacer insertion.

Guide RNA sequences were identified using Protospacer software (http://www.protospacer.com/) and cloned into pDC2-PK-Cas9-hDHFRyFCU as described [40]. Repair DNA plasmids were synthesised and cloned into pGEMT-Easy vector. For disruption of *ripr*, DNA strings were synthesised corresponding to bp 661–1006 and bp 1054–1398 of the endogenous *ripr* and were linked by either a recodonized 48 bp DNA sequence (pGEMT-PkRIPRrecodonized) or an XmaI site (pGEMT-PkRIPRXmaI). The triple HA tag with a stop codon was amplified by PCR using primer pair HAfor(XmaI)/HArevXmastop and plasmid pHH4PkRIPR-HA. The triple HA tag was inserted into pGEMT-PKRIPRXmaI resulting in pGEMT-PkRIPRko. Similarly, DNA strings for *cyrpa* were synthesised comprising endogenous DNA sequences 219–573 bp and 609–1109 bp being either linked by 36 bp of recodonized DNA sequence or an XmaI site and cloned into pGEMT-Easy vector resulting in vectors pGEMT-PkCyRPArecodonized and pGEMT-PkCyRPAXmaI. Finally, insertion of the triple HA-tag comprising a stop codon into the XmaI site generated plasmid pGEMT-PkCyRPAko.

20 μg of guide plasmid and 60 μg of EcoRI-linearized repair plasmids were transfected into nycodenz-purified PkA1-H.1 late schizonts. Transfected parasites were selected with 50 nM pyrimethamine for 3 days. Parasites derived from CRISPR/Cas9 transfection were analyzed by PCR.

For the generation of a DiCre-expressing PkA1-H.1 parasite line we generated the PkpSKIP_Pk47 vector (S8 Fig), by replacing the *crt* promoter in pSKIPFlox [41] with 859 bp of Pk*hsp86* promoter amplified from pDC2-PK-Cas9-hDHFRyFCU using primers Pkhsp86promFBglSac and Pkhsp86promrevXho. We then amplified 500 bp of the *pfs47* homologue in *P*. *knowlesi* (PKNH_1254100) using primer pair PK47HR1for2BglII / PK47HR1rev2SacII and cloned this fragment upstream of the Pk*hsp86* promoter using restriction enzymes BglII and SacII. We further amplified a second, 385 bp homology region from PKNH_1254100 using primer pair PK47HR2forEcoRV / PK47HR2rev2short and cloned this fragment into the EcoRV-digested vector downstream of the PbDT3’ UTR sequence.

A guide plasmid targeting PKNH_1254100 was generated by cloning annealed and phosphorylated primer pair pDC946For / pDC946Rev into pDC2-PK-Cas9-hDHFRyFCU. 20 μg of guide plasmid pDC946 was transfected together with 110 μg of BglII and BtgZ1-linearized PkpSKIP_Pk47 repair plasmid. Resulting transfectants were cloned by limiting dilution and analyzed by PCR and DNA sequencing. All work using DiCre recombinase-expressing *P*. *knowlesi* parasites was carried out on clone PkpSKIP9-10.

To flox and epitope tag the ORFs of Pk*ptramp*, Pk*css* and Pk*cyrpa* synthetic repair DNA plasmids were synthesized by GeneArt (Thermo Fisher Scientific). For the Pk*ptramp* repair plasmid (pMKPkptrampiKOrepair), 290 bp of promoter together with 28 bp of ORF sequence were used as homology region 1 followed by 44 bp of recodonized ORF sequence before the insertion of a triple HA-tag and a *loxPint* sequence based on the intron 4 sequence of Pk*dbpalph*a (S8 Fig), followed by the remaining 948 bp of recodonized ORF sequence, a *loxP* sequence after the stop codon, and 310 bp of 3’ UTR region functioning as homology region 2. The Pk*css* repair plasmid (pMKPkpcssiKOrepair) was designed of 300 bp of *css* promoter and 55 bp of 5’ ORF sequence functioning as homology region 1, 14 bp of recodonized DNA sequence, a triple HA-tag, a *loxPint* insertion, 1014 bp of recodonized ORF sequence, a *loxP* site after the stop codon and 311 bp of 3’ UTR sequence which functioned as homology region 2. For the Pk*cyrpa* repair plasmid (pMKPkcyrpaiKOrepair) DNA sequence corresponding to nucleotides 273–603 of *cyrpa* functioning in this plasmid as homology region was synthesized and is followed by recodonized ORF sequence (11 bp), *loxPin*t, the remaining 484 bp of C-terminal ORF sequence which was recodonized, a triple HA-tag, a stop codon, a *loxP* sequence and 323 bp of 3’UTR sequence. As before the 3’UTR sequence is used as the region in the homologous recombination DNA repair step following DNA cleavage by Cas9 nuclease.

We transfected PkpSKIP9-10 DiCre recombinase expressing parasites with 60 μg of repair plasmids together with 20 μg of each of two guide RNA plasmids (pMKPkptrampiKOrepair with pDC45+pDC982; pMKPkpcssiKOrepair with pDC89 + pDC1081; pMKPkcyrpaiKOrepair with pDC591+pDC1229). Locations of guide RNAs were chosen in order to direct DNA cleavage close to either of the two homologous recombination flanks (see S4 Fig). Transfected parasites were selected with 50 nM pyrimethamine for 4 days. Parasites derived from these CRISPR/Cas9 transfections were cloned by limiting dilution and validated by PCR and immunoblotting.

### Protein expression and purification

For recombinant PkPTRAMP and PkCSS expression, full-length, codon-optimized (for human expression) ORFs lacking the signal sequence were synthesized (encoding residues Val20 –Ala362 of PkCSS and residues Asp20-Phe296 of PTRAMP). The genes were cloned into the pHLsec vector [58] using KpnI and AgeI. Expi293F cells (Thermo Fisher Scientific) were transfected with these constructs and culture supernatant harvested after 6 days.

For PkRIPR expression, two gene fragments were synthesized, corresponding to PfRIPR fragments previously successfully expressed [19]: an N-terminal fragment (PkRIPR residues Lys287-Glu371) and a C-terminal fragment (residues Asp741-Asp850). The gene fragments were cloned into a modified pET15b vector (Novagen) and the resultant plasmid transformed into Shuffle cells (NEB). Expression was induced and the cells incubated at 30°C for 16 h, before harvesting and lysing of the cells by sonication.

Recombinant PkPTRAMP, PkCSS, and PkRIPR proteins were purified by affinity chromatography using nickel-nitrilotriacetic acid resin (Ni^2+^-NTA). The eluate was concentrated (Amicon Ultra centrifugal filter; MWCO 10 kDa) and gel filtered using a Superdex 200 16/60 column (PkPTRAMP and PkCSS) or a Superdex 75 16/60 column (PkRIPR; GE Healthcare), in 20 mM HEPES, 150 mM NaCl, pH 7.5.

### Antibody production

For anti-PkRIPR, anti-PkPTRAMP and anti-PkCSS antibodies, rabbit immunizations were carried out by Covalab in compliance with the UK Animals (Scientific Procedures) Act. Rabbit antibodies against PkRIPR were raised against the combination of two recombinant proteins as described above.

### Erythrocyte binding assays

Erythrocytes were washed in RPMI 1640 medium. 50 μl packed erythrocytes were added to 45 μg, 15 μg, 5 μg, 1.67 μg, or 0 μg of recombinant protein in 150 μl RPMI 1640 medium. Recombinant proteins tested were PkPTRAMP, PkCSS, PfMSP3 and full-length PfRH5 [16]. The samples were incubated at room temperature for 1 h with gentle shaking. Thereafter, samples were separated through dibutyl phthalate, washed and bound protein eluted with 20 μl of 1.5 M NaCl. Input protein and the eluate were analyzed by immunoblot probed with anti-His antibodies.

### Immunofluorescence assays

Indirect immunofluorescence assays (IFAs) were performed on purified schizonts, merozoites and early ring stages of PkRIPR-HA clone G4 as well as on mature schizonts of PkptrampiKO, PkcssiKO and PkcyrpaiKO clones. IFAs were carried out as described [59] using mAb 3F10 anti-HA, rabbit anti-PkAMA1, and mouse anti-PkRhopH2 antibodies and visualized using a Nikon Eclipse Ni microscope 100x Plan Apo λ NA 1.45 objective. Images were collected using an Orca Flash 4 digital camera controlled by Nikon NIS Elements AR 4.30.02 software.

### Invasion and growth inhibitory antibody assays (GIA)

Invasion assays for *P*. *falciparum* and *P*. *knowlesi* merozoites were carried out as described [28,60]; for *P*. *knowlesi*, a 3 μm filter was followed by a 2 μm filter (Whatman) to release merozoites. Merozoites were added rapidly to erythrocytes and any additives, at 1% final hematocrit. Additives were anti-BSG mAb MEM-M6/6 (ThermoFisher) and soluble BSG [16]. The final heparin concentration used to inhibit invasion was 71.4 mU/μl for *P*. *falciparum*, and 125 mU/μl for *P*. *knowlesi*. After 12 h, cells were washed in PBS, stained with SybrGreen and analyzed by flow cytometry.

For invasion inhibition assays with anti-PkRIPR, anti-PkCSS, and anti-PkPTRAMP antibodies, synchronized populations of schizont-stage PkA1-H.1 parasites were added to erythrocytes in the presence of a serial dilution of purified IgG from 2 mg/ml. As control, nonspecific rabbit IgG was used (Sigma). After 18 h, cells were stained with SybrGreen and parasitemia was analyzed by flow cytometry.

Growth inhibition assays using anti-BSG and anti-DARC antibodies were carried out with *P*. *falciparum* 3D7 and *P*. *knowlesi* A1-H.1 lines, and *P*. *vivax* field isolates. The antibodies used were anti-BSG mAbs MEM-M6/6 and TRA-1-85 (R&D Biosystems); anti-human CD147 goat polyclonal antibody (R&D Biosystems); anti-DARC nanobody CA111 (a kind gift from Olivier Bertrand and Yves Colin); non-specific mouse IgG (Sigma); and non-specific goat IgG (R&D Biosystems). *P*. *falciparum* and *P*. *knowlesi* GIAs were carried in complete RPMI 1640+AlbumaxII and supplemented with 10% horse serum, at 2% hematocrit. Synchronized populations of schizont-stage parasites were incubated with a serial dilution of antibodies from 20 μg/ml; after 40 h, cells were fixed, stained with Hoechst 33342 and parasitemia was measured by FACS.

*P*. *vivax* invasion assays were carried out with isolates taken from patients with acute infection from communities close to Iquitos city in Peru. Ten ml samples of blood were collected from patients with single *P*. *vivax* malaria infections and a parasite density of >0.1%. Platelets and leukocytes were depleted from samples with ≥80% parasites at the ring or trophozoite stages, using a CF11 column [61]. After *P*. *vivax* maturation invasion assays were performed in 96-well plates as previously described [62,63] using reticulocyte-enriched (>50%) erythrocyte preparations. Reticulocytes were purified from blood samples of hemochromatosis patients within 48 h of collection. These samples were typed for the Duffy phenotype (Fy) using standard serological methods (DiaMed-ID Micro Typing Systems, DiaMed), and leukocytes removed using filters (Fresenius Kabi), before enrichment of reticulocytes by differential centrifugation [64] followed by Percoll gradient [62]. Antibodies were tested at 10 μg/ml for invasion assays. Assays were set up at a 1:6 ratio of parasite to reticulocyte enriched RBCs (as described [63]) and invasion inhibition was measured in paired samples using the same parasite isolates (a total of 10). For valid invasion assays, the parasitemia of the untreated control was ≥0.5% (S1 Table). Level of invasion was analyzed by microscopy and calculated as the percentage of ring stage parasite-infected erythrocytes 24 h post-invasion, counting >9000 erythrocytes. Statistical analysis was performed using Wilcoxon matched-pairs signed-rank test through GraphPad Prism7.

### Immunoprecipitations and immunoblotting

PkRIPR immunoprecipitations (IPs) were performed using transgenic PkRIPR-HA G4 and wild type PkA1-H.1 parasites. Purified schizonts were dissolved in lysis buffer (1% NP40, 150 mM NaCl, 50 mM Tris pH 8.0, 5 mM EDTA, complete protease inhibitors) and concentrated culture supernatants were diluted 1:1 in lysis buffer lacking NP40. Supernatants were precleared using unconjugated agarose beads for 2 h before incubation with anti-HA conjugated agarose beads overnight at 4°C. Two different affinity matrix products were used: HA Tag IP/Co-IP Kit (Pierce) and anti-HA 3F10-agarose beads (Roche). Beads were washed with 10 mM Tris-HCl, pH 7.5, 300 mM NaCl, then PBS, and eluted in 100 mM glycine-HCl pH 2.5. Eluates were neutralized and concentrated using Vivaspin 5kDa MWCO columns, separated on 10% Bis-Tris NuPAGE gels, and stained with Colloidal Blue. Similarly, immunoprecipitations using anti-HA 3F10-agarose beads (Roche) and schizont lysates from PkptrampiKO, PkcssiKO and PkcyrpaiKO were conducted.

### Mass spectrometry and analysis

Band slices excised from NuPAGE gels covering the whole separation profile were washed, reduced and alkylated before digestion with 2 μg/ml trypsin, overnight. Peptides were analyzed by nano-liquid chromatography tandem mass spectrometry (nano-LC-MS/MS) as described [56]. Raw data were processed using Maxquant 1.3.0.5 and Perseus 1.4.0.11 and searched against the *P*. *knowlesi* fasta database on PlasmoDB release 25 (immunoprecipitations from PkRIPR-HA clone G4) and PlasmoDB release 26 (immunoprecipitations from PkptrampiKO, PkcssiKO and PkcyrpaiKO) and human reference proteome (UNIPROT) using Andromeda. A decoy database of reversed sequences was used to filter the results, removing false positives, at a false detection rate of 1%. Label free quantitation was achieved using iBAQ.

## Supporting information

S1 Fig Box-and-whiskers plot of growth inhibition assay (GIA) data of *P*. *vivax* field isolates. The isolates were incubated with media control or treated with anti-BSG (MEM M6/6 or polyclonal antibody) or non-specific mouse or goat IgGs. Whiskers represent min and max values. Median values of control to anti-BSG antibody treatments are significantly different (**, p = 0.0078 and p = 0.0021); no significant difference was seen comparing control to non-immune IgG treatment. Statistical analysis was performed using Wilcoxon matched-pairs signed-rank test in GraphPad Prism7.
(PDF)

S2 Fig Alignments of CyRPA sequences from 14 *Plasmodium* species (A) and RIPR sequences from 18 *Plasmodium* species (B). Similar residues (as calculated using MultAlin) are shown in color. The color corresponds to the residue type (with H,K,R, green; D,E, red; S,T,N,Q, maroon; C, green; P,G, orange; A,V,L,I,M, pink; F,Y,W, blue). The secondary structural elements of PfCyRPA are shown above in (A), with beta strands represented as arrows and helices as coils. The ten predicted EGF domains of RIPR are indicated (B).
(PDF)

S3 Fig Relative abundance of *rh5*, *cyrpa* and *ripr* mRNA transcripts throughout the life cycle of *P*. *falciparum*, *P*. *knowlesi*, and *P*. *vivax*. The log2 ratio of mRNA abundance relative to a pooled reference sample is plotted for 24 time points (*P*. *falciparum*), seven time points (*P*. *knowlesi*), or nine time points (*P*. *vivax*) (taken from [34]). Colors as follows: *cyrpa*, blue; *ripr*, red; *rh5*, green.
(PDF)

S4 Fig Design and genetic validation of inducible knockout parasites PkptrampiKO, PkcssiKO and PkcyrpaiKO. (A) Schematic of inducible knockout design for *ptramp* and *css* genes in *P*. *knowlesi*. ORFs are depicted as dark grey box with predicted signal peptide (SP) sequence outlined. Also indicated are the homology regions (HR) used in the repair plasmids. We used guide plasmids pDC45 and pDC982 to target *ptramp* and guide plasmids pDC89 and pDC1081 to target *css*. One guide RNA sequence is homologous to a DNA region close to the signal peptide sequence of either gene and the other targeted a region close to the stop codon. Turquoise box represents the introduction of a triple HA tag after the predicted signal peptide cleavage site. Positions of the *loxP* sequences are indicated. The *loxPint* sequence is derived from the *Pkdbpalpha* intron 4 sequence. Schematic of floxed *ptramp* and *css* loci before and after excision is shown. (B) PCR screens of wild type (wt = PkpSKIP9-10) and two clones of PkptrampiKO and of PkcssiKO are shown. Primer positions are indicated in schematic A, with the green primer sequences homologous to the *css* locus. Primer combinations are indicated on the top of each image with primer name and sequence listed in S2 Table and DNA size standards are shown on the left (in kb). (C) Schematic of PkcyrpaiKO design. Endogenous ORF of *cyrpa* is shown in grey as two exons connected by one intron. HR regions used in the repair plasmid are indicated as are the homologous regions of the guide plasmids pDC591 and pDC1229. In the repair plasmid we replaced the endogenous intron with the *loxPint* sequence and inserted a triple HA-tag after the stop codon followed by a *loxP* sequence. Schematics of the floxed locus before and after excision are given. (D) PCR screen of wild type (wt = PkSKIP9-10) and two PkcyrpaiKO clones. Primer positions are indicated in schematic C and primer combinations for PCR amplification are shown at the top of each lane. Primer names and sequences are listed in S2 Table. DNA size standards are shown on the left (in kb).
(PDF)

S5 Fig Indirect immunofluorescence assay of inducible knockout parasites PkptrampiKO, PkcssiKO and PkcyrpaiKO. We used anti-HA antibodies (green) to localize HA-PTRAMP, HA-CSS and CyRPA-HA in the respective transgenic parasite lines (indicated on the left of each image row) and co-localized this signal with antibodies to the micronemal marker AMA1 (red, top panels) but not with antibodies to the rhoptry bulb marker RhopH2 (red, bottom panels). Nuclei are stained with DAPI (blue). Scale bar = 2 μm.
(PDF)

S6 Fig Purification of recombinant proteins corresponding to PkCSS, PkPTRAMP and two fragments of PkRIPR (Lys287-Glu371 and Asp741-Asp850) used to produce antibodies. After Ni^2+^-NTA chromatography, proteins purified further by gel filtration (Superdex 75 column for the PkRIPR fragments and Superdex 200 column for PkCSS and PkPTRAMP). (A) UV traces of gel filtration elution are shown at the top, and Coomassie blue-stained SDS-PAGE gels of selected fractions at the bottom. Arrows indicate the expected protein bands: PkRIPR Lys287-Glu371 (~12 kDa), PkRIPR Asp741-Asp850 (~15 kDa), PkCSS (~43 kDa), and PkPTRAMP (~36 kDa). Recombinant PkCSS and PkPTRAMP contain putative N-linked glycan sites and glycosylation might account for the decreased mobility in SDS-PAGE. (B) Immunoblots of total PkA1-H.1 lysate separated by SDS-PAGE and probed with either preimmune or immune rabbit serum. Rabbits were injected with recombinant proteins as indicated at the bottom of each immunoblot. Molecular mass standards are shown to the left of each blot. Bands corresponding to expected sizes of the native proteins are indicated by arrow heads.
(PDF)

S7 Fig Plasmid map for CRISPR/Cas9 vector pDC2-PK-Cas9-hDHFRyFCU1. *P*. *knowlesi* specific 5’UTR regions of *hsp86*, *ef1alpha* and U6 were selected to drive transcription of *cas9* nuclease, selection marker *hdhfr*:*yfcu* and the protospacer. Total vector size is 12.464 kb (A). (B) Decorated schematic of 240 bp of vector sequence including U6 terminator, guide RNA sequence with BbsI cloning sites (arrow heads) for protospacer insertion.
(PDF)

S8 Fig Plasmid map of PkpSKIP_pk47. Plasmid is derived from pSKIP vector [41] with *P*. *knowlesi*-specific homology regions to direct the insertion of the DiCre recombinase cassette into the genome (A). Schematic of *loxPint* sequence derived from the 83 bp intron-4 sequence of *P*. *knowlesi dbp alpha* with the insertion of a *loxP* site without affecting RNA branching points.
(PDF)

S1 Table Table of *P*. *vivax* isolate names, their treatment and their parasitemias (untreated). The treatment conditions used for each isolate, which are reported in Fig 1E, are indicated by tick marks.
(PDF)

S2 Table Primer name and sequences used in this study. Restriction enzyme sites within the oligonucleotide DNA sequence are listed on the right.
(PDF)

S1 Dataset Mass spectrometry data tables for anti-HA co-immunoprecipitation experiments using PkRIPR-HA parasites. (A) Table of proteins detected by LC-MS/MS using parasite lysate and culture supernatant with anti-HA affinity matrix (Roche). Immunoprecipitations from wild type PkA1-H.1 or PkRIPR-HA parasites were analysed by LC-MS/MS (three technical replicas) ordered by highest peptide abundance (iBAQ). (B) Table of proteins detected by LC-MS/MS using parasite lysate and culture supernatant from wild type and PkRIPR-HA parasites with Pierce HA-tag IP/Co-IP kit (Thermo Fisher Scientific) ordered by highest peptide abundance (iBAQ). Analysis was conducted using Maxquant 1.3.0.5 and Perseus 1.4.0.11 searched against the *P*. *knowlesi* fasta database (PlasmoDB release v25). IBAQ quantifications for immunoprecipitations from PkRIPR-HA culture supernatant are highlighted in green, from PkRIPR-HA schizont lysates in grey and from PkA1-H.1 schizont lysates or culture supernatant in white. Three top scoring proteins with high peptide abundance in the transgenic parasite line but minimal or no peptides detected in the wild type parasites are highlighted in yellow. iBAQ are log_10_ values.
(XLSX)

S2 Dataset Mass spectrometry data tables for anti-HA co-immunoprecipitation experiments using PkptrampiKO, PkcssiKO and PkcyrpaiKO parasites. Immunoprecipitations from purified schizonts of wild type (PkpSKIP 9–10) and PkptrampiKO, PkcssiKO and PkcyrpaiKO parasites were analysed by LC-MS/MS (three technical replicas) and ordered by highest peptide abundance (iBAQ). iBAQ are log_10_ values. Analysis was conducted using Maxquant 1.3.0.5 and Perseus 1.4.0.11 searched against the *P*. *knowlesi* fasta database (PlasmoDB release v26). Three top scoring proteins with highest peptide abundance in PkptrampiKO and PkcssiKO parasites are highlighted in yellow; PkCyRPA is highlighted in orange.
(XLSX)
