# Supplementary figures and images for: Divergent roles for the RH5 complex components, CyRPA and RIPR in human-infective malaria parasites
Source: PLoS Pathog. 2019 Jun 11;15(6):e1007809. doi: 10.1371/journal.ppat.1007809 (PMC6588255; doi:10.1371/journal.ppat.1007809)

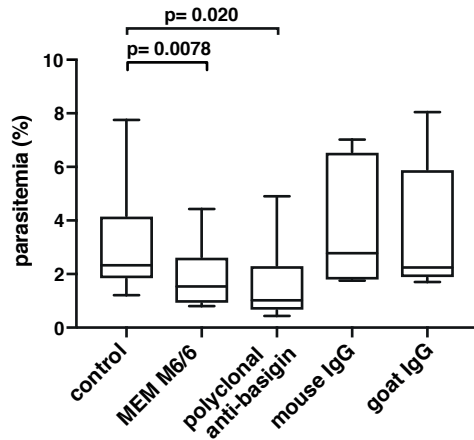

Supplement: S1 Fig — The isolates were incubated with media control or treated with anti-BSG (MEM M6/6 or polyclonal antibody) or non-specific mouse or goat IgGs. Whiskers represent min and max values. Median values of control to anti-BSG antibody treatments are significantly different (**, p = 0.0078 and p = 0.0021); no significant difference was seen comparing control to non-immune IgG treatment. Statistical analysis was performed using Wilcoxon matched-pairs signed-rank test in GraphPad Prism7. (PDF) [file ppat.1007809.s001.pdf]

*P. falciparum*

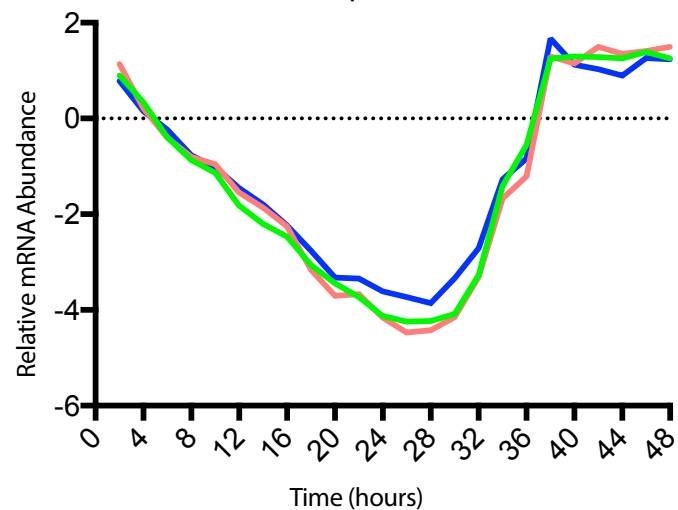

*P. knowlesi*

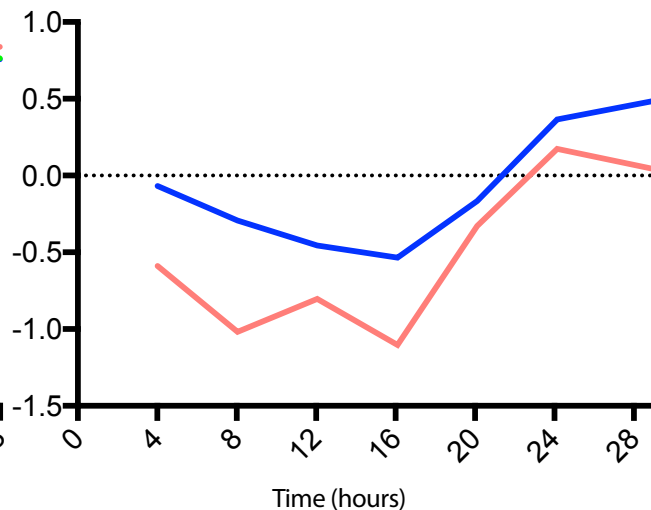

*P. vivax*

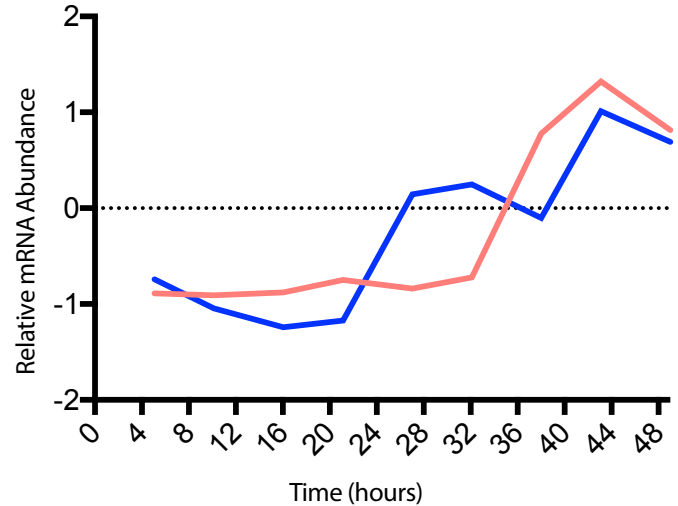

- RH5
- RIPR
- CyRPA

Supplement: S3 Fig — The log2 ratio of mRNA abundance relative to a pooled reference sample is plotted for 24 time points (P. falciparum), seven time points (P. knowlesi), or nine time points (P. vivax) (taken from [34]). Colors as follows: cyrpa, blue; ripr, red; rh5, green. (PDF) [file ppat.1007809.s003.pdf]

**A**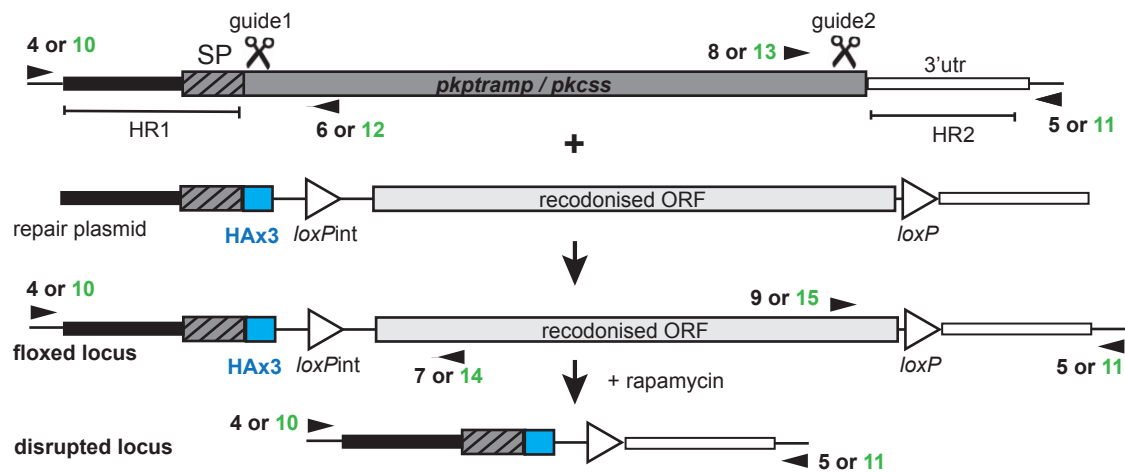**B**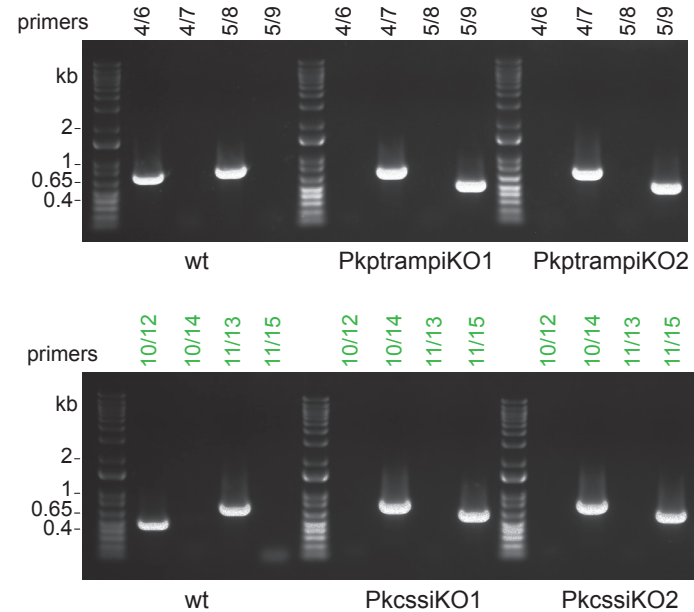**C**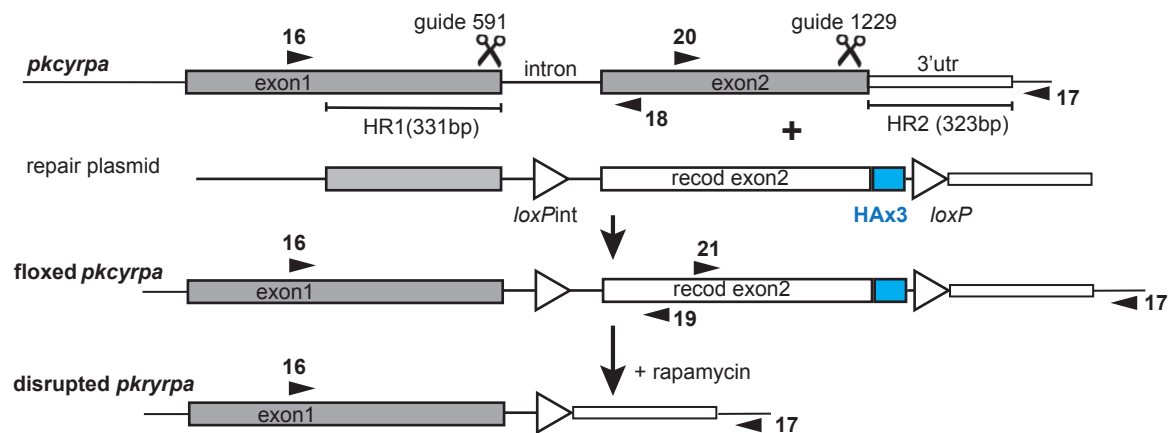**D**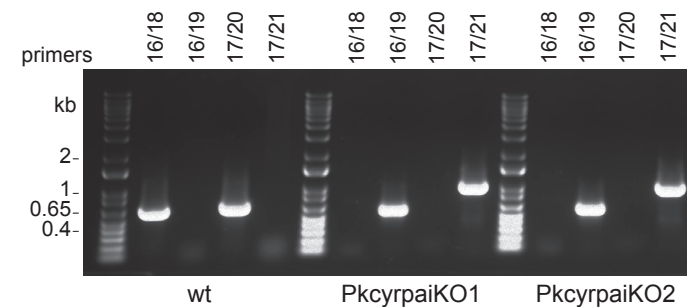

Supplement: S4 Fig — (A) Schematic of inducible knockout design for ptramp and css genes in P. knowlesi. ORFs are depicted as dark grey box with predicted signal peptide (SP) sequence outlined. Also indicated are the homology regions (HR) used in the repair plasmids. We used guide plasmids pDC45 and pDC982 to target ptramp and guide plasmids pDC89 and pDC1081 to target css. One guide RNA sequence is homologous to a DNA region close to the signal peptide sequence of either gene and the other targeted a region close to the stop codon. Turquoise box represents the introduction of a triple HA tag after the predicted signal peptide cleavage site. Positions of the loxP sequences are indicated. The loxPint sequence is derived from the Pkdbpalpha intron 4 sequence. Schematic of floxed ptramp and css loci before and after excision is shown. (B) PCR screens of wild type (wt = PkpSKIP9-10) and two clones of PkptrampiKO and of PkcssiKO are shown. Primer positions are indicated in schematic A, with the green primer sequences homologous to the css locus. Primer combinations are indicated on the top of each image with primer name and sequence listed in S2 Table and DNA size standards are shown on the left (in kb). (C) Schematic of PkcyrpaiKO design. Endogenous ORF of cyrpa is shown in grey as two exons connected by one intron. HR regions used in the repair plasmid are indicated as are the homologous regions of the guide plasmids pDC591 and pDC1229. In the repair plasmid we replaced the endogenous intron with the loxPint sequence and inserted a triple HA-tag after the stop codon followed by a loxP sequence. Schematics of the floxed locus before and after excision are given. (D) PCR screen of wild type (wt = PkSKIP9-10) and two PkcyrpaiKO clones. Primer positions are indicated in schematic C and primer combinations for PCR amplification are shown at the top of each lane. Primer names and sequences are listed in S2 Table. DNA size standards are shown on the left (in kb). (PDF) [file ppat.1007809.s004.pdf]

**A**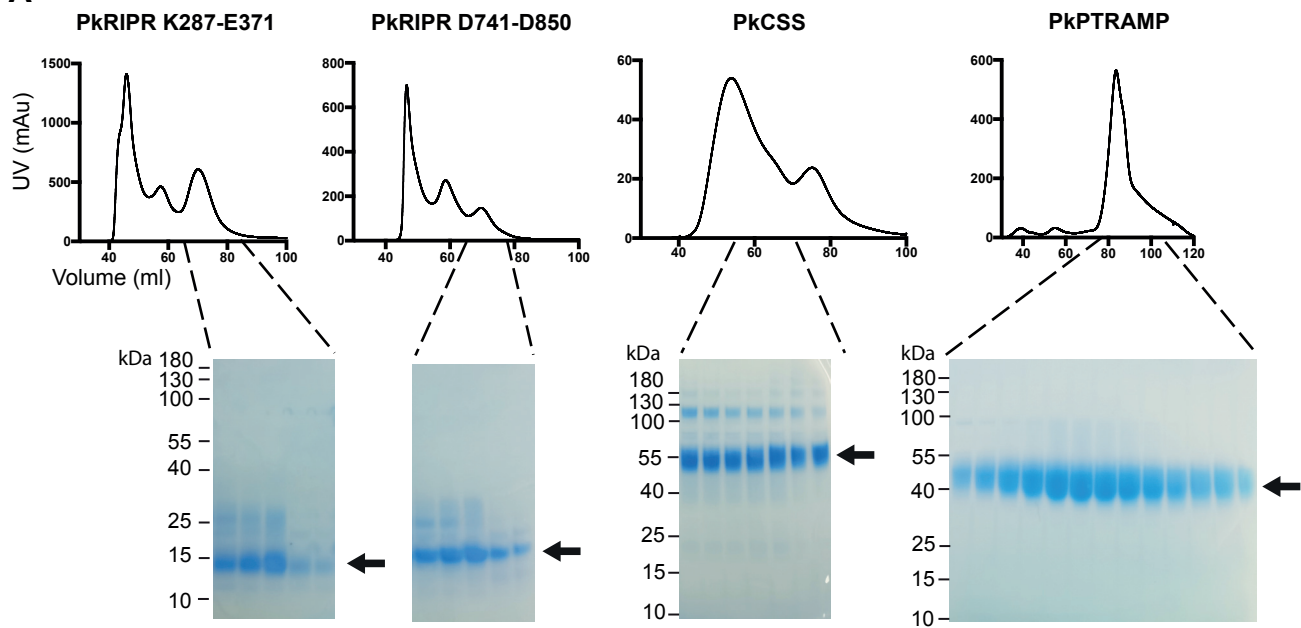**B**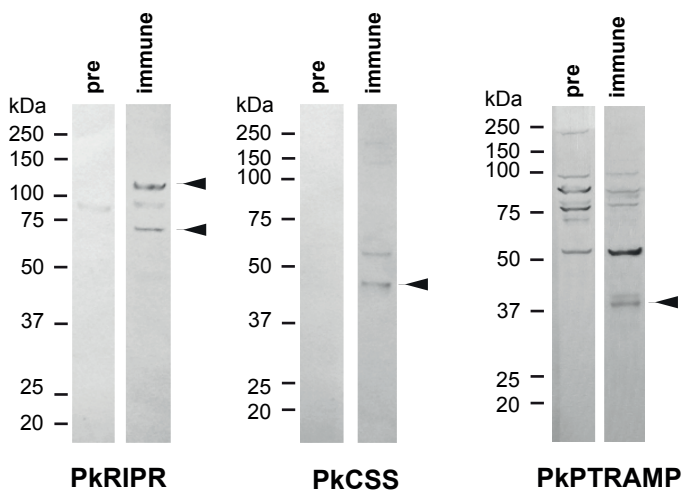

Supplement: S6 Fig — After Ni2+-NTA chromatography, proteins purified further by gel filtration (Superdex 75 column for the PkRIPR fragments and Superdex 200 column for PkCSS and PkPTRAMP). (A) UV traces of gel filtration elution are shown at the top, and Coomassie blue-stained SDS-PAGE gels of selected fractions at the bottom. Arrows indicate the expected protein bands: PkRIPR Lys287-Glu371 (~12 kDa), PkRIPR Asp741-Asp850 (~15 kDa), PkCSS (~43 kDa), and PkPTRAMP (~36 kDa). Recombinant PkCSS and PkPTRAMP contain putative N-linked glycan sites and glycosylation might account for the decreased mobility in SDS-PAGE. (B) Immunoblots of total PkA1-H.1 lysate separated by SDS-PAGE and probed with either preimmune or immune rabbit serum. Rabbits were injected with recombinant proteins as indicated at the bottom of each immunoblot. Molecular mass standards are shown to the left of each blot. Bands corresponding to expected sizes of the native proteins are indicated by arrow heads. (PDF) [file ppat.1007809.s006.pdf]

A

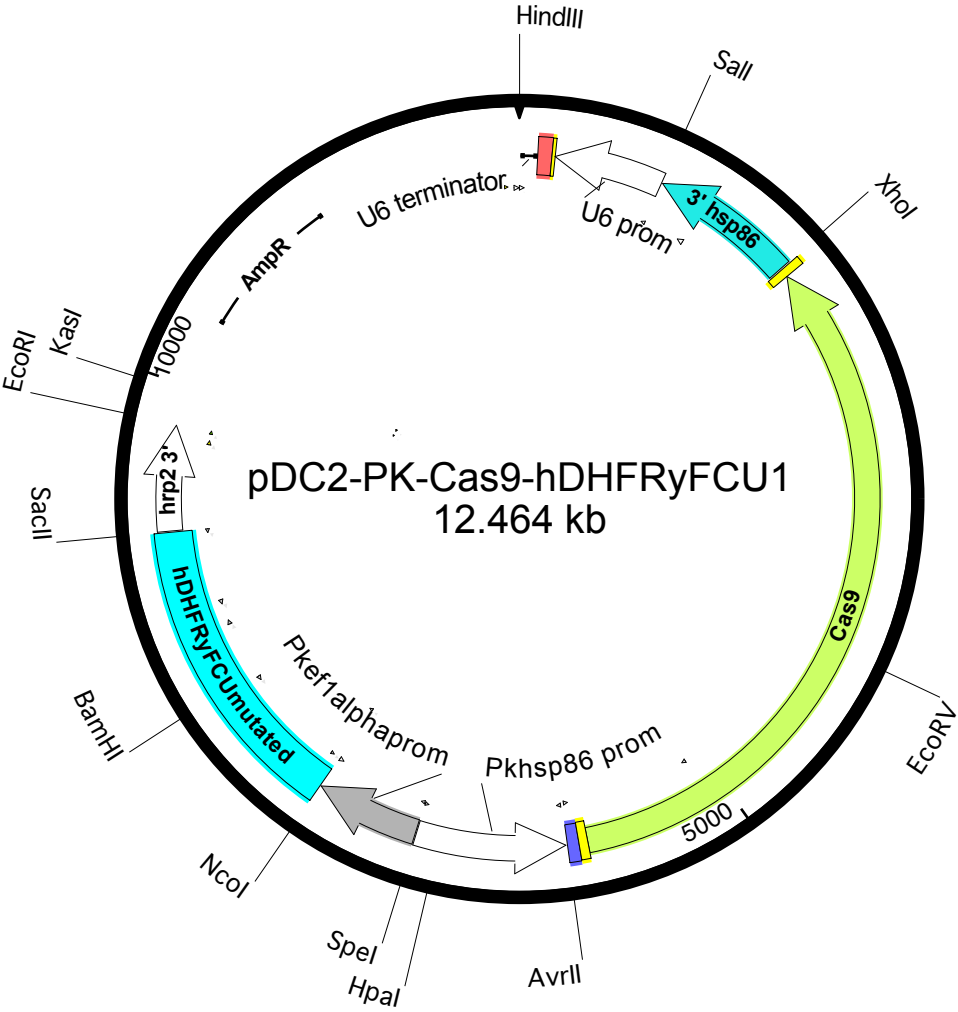

B

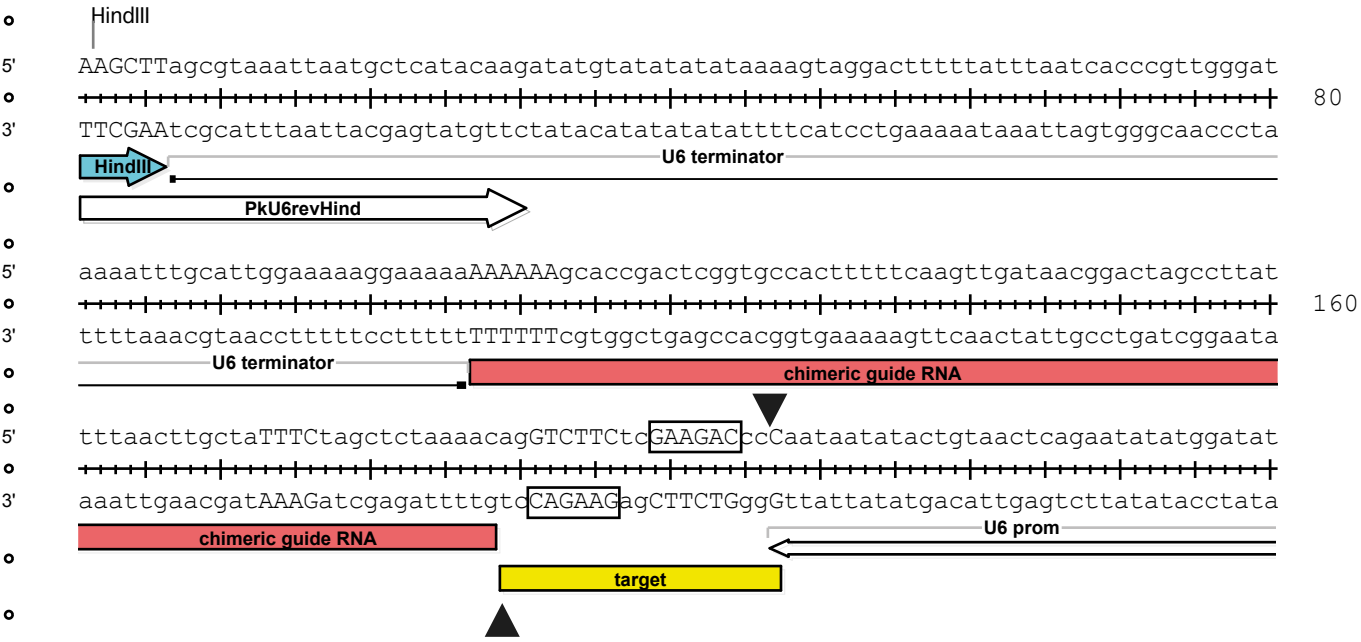

Supplement: S7 Fig — P. knowlesi specific 5’UTR regions of hsp86, ef1alpha and U6 were selected to drive transcription of cas9 nuclease, selection marker hdhfr:yfcu and the protospacer. Total vector size is 12.464 kb (A). (B) Decorated schematic of 240 bp of vector sequence including U6 terminator, guide RNA sequence with BbsI cloning sites (arrow heads) for protospacer insertion. (PDF) [file ppat.1007809.s007.pdf]

A

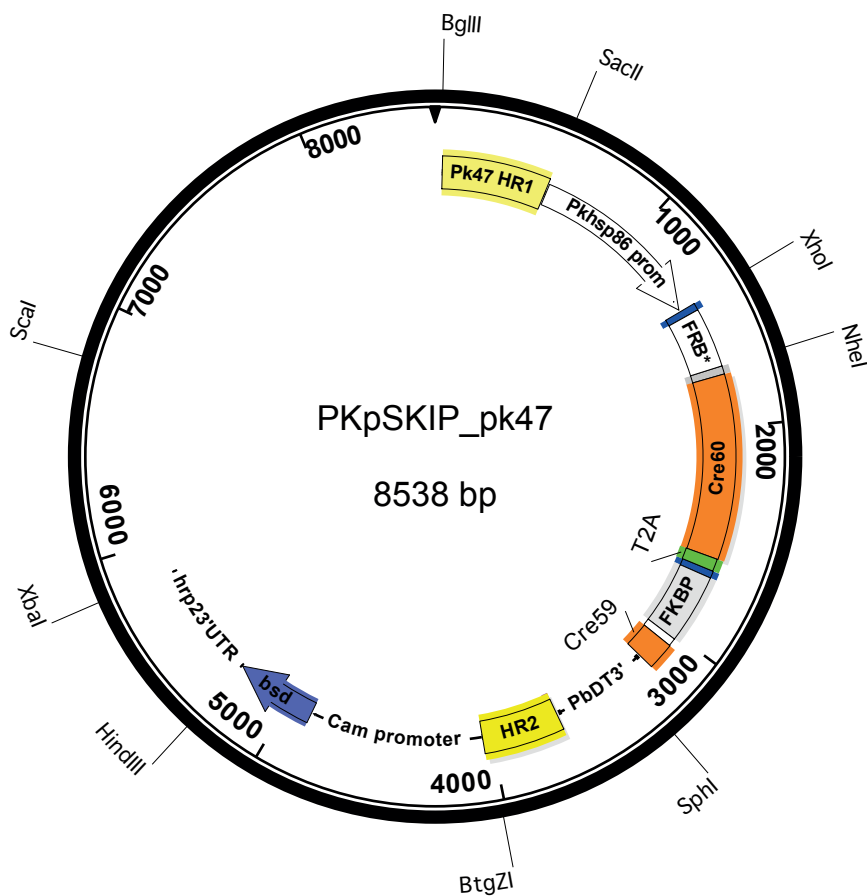

B

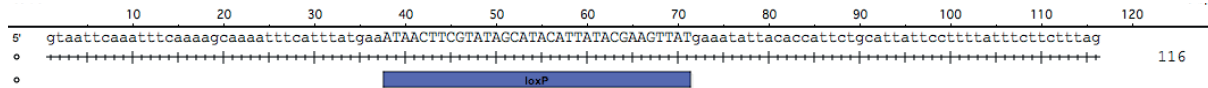

Supplement: S8 Fig — Plasmid is derived from pSKIP vector [41] with P. knowlesi-specific homology regions to direct the insertion of the DiCre recombinase cassette into the genome (A). Schematic of loxPint sequence derived from the 83 bp intron-4 sequence of P. knowlesi dbp alpha with the insertion of a loxP site without affecting RNA branching points. (PDF) [file ppat.1007809.s008.pdf]
