# Supplementary material for: Divergent roles for the RH5 complex components, CyRPA and RIPR in human-infective malaria parasites
Source: PLoS Pathog. 2019 Jun 11;15(6):e1007809. doi: 10.1371/journal.ppat.1007809 (PMC6588255; doi:10.1371/journal.ppat.1007809)
Supplement: S2 Fig — Similar residues (as calculated using MultAlin) are shown in color. The color corresponds to the residue type (with H,K,R, green; D,E, red; S,T,N,Q, maroon; C, green; P,G, orange; A,V,L,I,M, pink; F,Y,W, blue). The secondary structural elements of PfCyRPA are shown above in (A), with beta strands represented as arrows and helices as coils. The ten predicted EGF domains of RIPR are indicated (B). (PDF) [file ppat.1007809.s002.pdf]

A. CyRPA sequence alignment

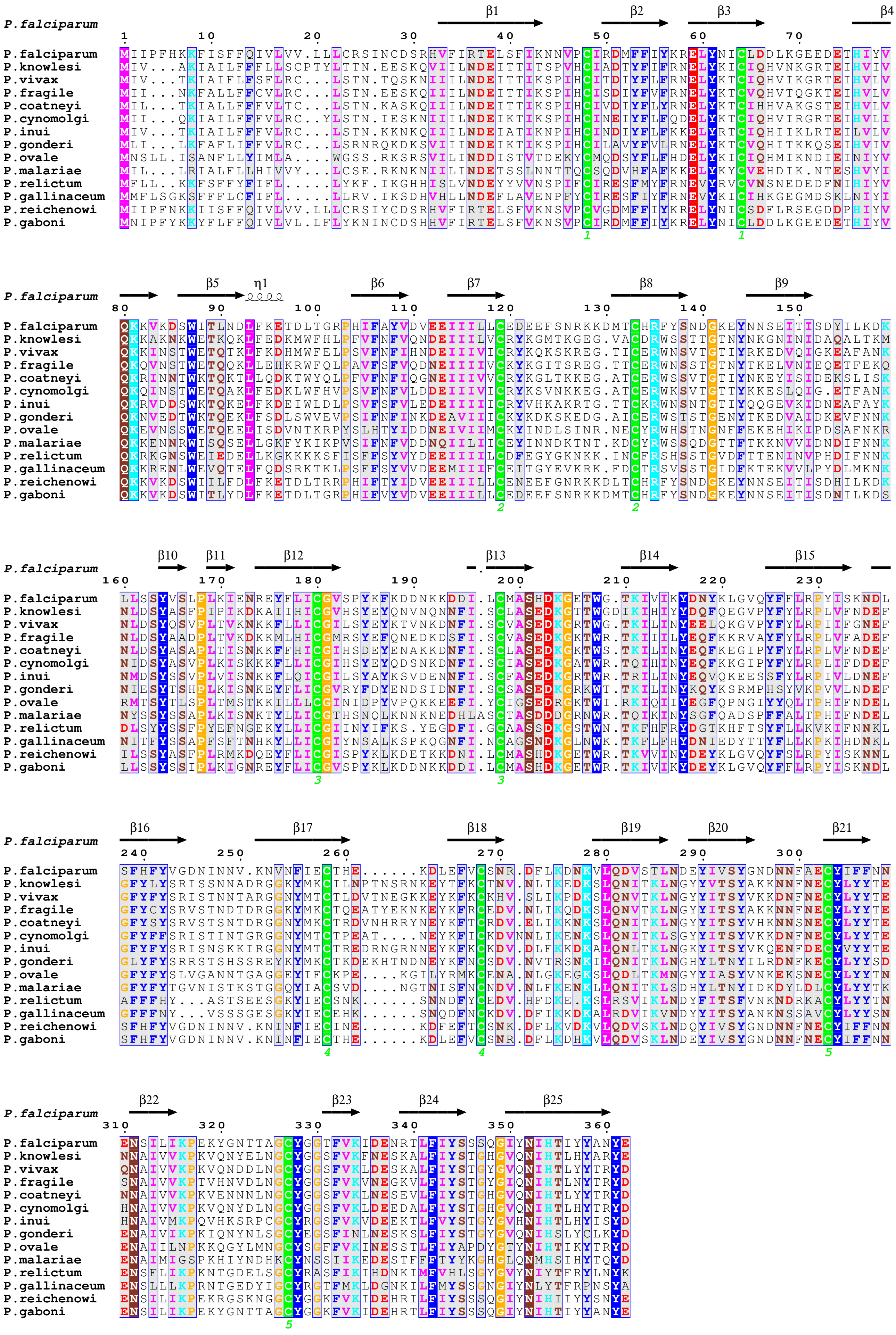

B. RIPR sequence alignments

```
                                     1       10        20        30        40        50        60
P.falciparum    .....................MFRIFF.TLLIIILIKKTSAIDLIEGIFYEKN..EIDKLTFSLDHRVRDNLKTDLILNNNGENDYAYLN
P.knowlesi      ....................MNCWVCV..LLLLVGVRKSRANVVEDDIYYQKE..EVDKLTFSLDHRVRDNSIEEWPEDTNGENAYGYIN
P.vivax         ....................MKCRVCILLLLLLVGVRRGRANVVEDDIFYQKG..EVEKLTFSLDHRVRDKSKKERLDYTNGENAYAYVD
P.gallinaceum   ....................MLFFHLF..FFFIVLKKKILSLDIVDRIFYDVD..DLKGIDFSLDYRNREKLATQNIENNNGEQSYGYSH
P.relictum      ....................MWFFYLF..YLFTVLEKSFFSLDIVDRIFYQTN..EVRNLNFSLDYRNSEKLATDDPIYDNGEQLYGYTH
P.gaboni        .....................MFRIFF.TLLIIVLIKKTSTIDLIDGIFYEKN..EINKLTFSLDHRVRDNLKTDSILNNNGENDYAYLN
P.reichenowi    .....................MFRIFF.TLLIIVLINKISAIDLIEGIFYEKN..EIDKLTFSLDHRVRDNLKTDLILNNNGENDYAYLN
P.ovale         ....................MAFKWLS.FMFSFLLMRTVSGIDMIDDLYYQKD..EINKLTFSLDHRVRDKSKKENVGYTNGENAYAYMS
P.malariae      MSAARFVGIYHSQFSRETKKMAFKWLL..ISVIVFGKKVLTLNIVNSIFYQKD..EINNLTFSLDHRAREKAERAKLHFKNGEISYVYSD
P.coatneyi      ....................MNCWVCV..LLLLVGVRRGTANVVEDDIFYQKG..EVDKLTFSLDHRVRDKSMNEILEYTNGENAYAYVD
P.cynomolgi     ..........................................................................................
P.fragile       ....................MKCWVCV..LLLLVGMRKGRAIVVEDDIYYQKE..ELDKLTFSLDHRVRDKSMDDKVEYTNGENAYGYVD
P.inui          ....................MKCRVLV..LLLLVGVRRGRANIVEDDIFYQKE..EVEKLSFSLDHRVRDKSTKETVGHTNGVNAYAYVD
P.gonderi       ....................MKIRMGL.LLLLITGARKAKGNIVEDDIYYQNE..EIDKLMFSLDHRVRDKSKKEEVKYVNGENAYGYTN
P.berghei       MMKKKI............ITNLIIIIF.GLILFKCSIRGRKDITLSKANHNDD..NNSNILFSLNYKIRDKLHKGKIIHADGINTYGYKH
P.yoelii        MMKKIIM...........ITNLIIIIL.GLILFNCSWCGSKDIILNKTNNDDDNNNNNNVLFSLNYKIRDKIHKGKIIHADGIDSYGYKQ
P.chabaudi      MMKRKI............TTKSFITIF.GFILFNLSWCGTKDNILNE........NNNNILFSLNYKIRDKLYKGKIIYSDGIKTYGYKQ
P.vinckei       MMKRKI............TTKSFITIF.GFILFNLSWCGSKDNILSE........NNNNILFSLNYKIRDKLHKGKILYADGIKTYGYKK

                  70        80        90       100         110       120
P.falciparum    KYVYTILNR.DSTEKIKTFFSHNKDMKSCDYFISKEYNSSDK..TNQICYKKTFCGVVIPNSEEI..............K..........
P.knowlesi      KAITKIFRV.TSEEEKKNFFSLNKDLKTCDYIIEKGSLKTDL..KNNKCYKKIVCG.VIPHANYL..............Q.GES......
P.vivax         KAIAKVFTL.TSEAEIKKFFSLNKDVKTCDYLIAQGSLKKDL..QKNKCYRKTFCG.VIPHREYI..............PGGGS......
P.gallinaceum   KSVEALLPI.SAPEKIREYFSESSDKNSCDYFITQENFFSSD..KKEKCINNTFCG.IIPKNAHI.........................
P.relictum      KSLENIIPI.RSHDKIKEYLSDIVDKNSCAYFITQEN.SSSE..LKEKCMNKHFCG.ILPKNAHI.........................
P.gaboni        KYVYTILNR.NSTEKIKTFFSYNKDIKSCDYFISKQYNSNDK..KNEICYKKTFCGVVLPNSEDI..............K..........
P.reichenowi    KYVYTILNR.DSTEKIKTFFSHNKDMKSCDYFISKQYNSSDK..TNQICYKKTFCGVVIPNSEEI..............K..........
P.ovale         KGISAILTL.TSENEMKNFLSLNEELKYCNFLLSHNELTKDKNVKNHKCYKKTFCG.IIPNTEIIMNTNEGNPVRGNPVR.GNT......
P.malariae      NGISSIFTL.TAEKDIKEFFSLNEDIKSCDYFITKGGLLNRE..KKHKCFRETFCG.IVPNFKYI..............K..........
P.coatneyi      KEIAKVFTL.TSETEIKNFFSLNKDLKTCDYLIAKGSLRTDV..KNNKCYKKIFCG.VIPHTKYI..............Q.GGS......
P.cynomolgi     ...AKVFTLATSEAEIKKFFSLNKDEKTCDYLIAKGSFMTNL..STNKCYKKTFCG.VIPHDDYI..............N.EES......
P.fragile       KAITKVFTL.TSEAEIKKFFSLNKDVKTCDYLIAQGSLKKDL..TNNKCYRKTFCG.VIPHTDYI..............QEGSM......
P.inui          KAIAKVFPL.ISEQEIKKFFSLNKDVKTCDYQIGKGSFKNYL..KNNSCYRKIFCG.VIVDDKDI..............R.GES......
P.gonderi       KGITKVFNV.YAEKEIKNFFRMSKDPSTCDHNISLGTVENRV..IDQICYKREFCG.IIAPEEYV..............KVDTTLDWYMR
P.berghei       KGVSIVIPL.VHENKIKDFFSVSDDENSCSYYIYQHGVSTNK..YSIKCYQNNFCG.ILPNNKYI.........................
P.yoelii        KGVSTVIPL.IHESKIKDFFSINDDEQTCNYHIYKHGISNNK..YNIKCYQNNFCG.LLPNNKYI.........................
P.chabaudi      KGVSIVIPL.IYENKIKDFFSVSDDERSCGYHIYEYGISNNK..YNYKCYQNNFCG.IIPNNKYI.........................
P.vinckei       KGVSIVIPL.IYENRIKDFFSVSDDERSCGYHIYEYGILNNK..YSYKCYQNNFCG.IIPNNKYI.........................

               130        140      150       160        170        180       190        200       210
P.falciparum    TNKITND.KLYCAHFNSTHIIIYYISQPLLLEPHVVYEET.FFEKGKNDQINCQGMYISLRSVHVHT..HNAILQQETLTYIKNLCDGKN
P.knowlesi      KSKEGKE.LTHCAYMDDTNILIYHLGQPQLLKPNVMYQEI.FFQESEKGVINCHGMDISLRYVGIHTNGNNNCIQDYLHDHLKNLCNGKK
P.vivax         SNKRESD.LIHCAYMNDTHIVIYHVGRPHLLKPNVVYEEI.FFQESEKGVISCHAMNINLRYVGIHTSGNNTCIQDYLQEHLKELCNEKK
P.gallinaceum   SEENKND.KMYCAHINRSHVIVYSVGQPVVVEPHMILQEL.FFEREKNGIISCHNSVIDIRYINVHT..QNGCLQENILSNIMETCNDKR
P.relictum      SEETKNN.RIYCAHINKSYIIIYSLGEPIVVEPNVVFQEL.FFEKEKKGILDCHGLTIDIRLINIHT..QHGCLQDKLLNNIMSECNEKS
P.gaboni        TNKIAND.KLYCAHFNSTHIIIYYISQPLLLEPNVVYEET.FFEKGKHDQINCQGMYISLRSVYVHT..DNTILQQETLTYIKNLCDGKN
P.reichenowi    TNKITND.KLYCAHFNSTHIIIYYISQPLLLEPDVVYEET.FFEKGKHDQINCQGMYISLRSVHVHT..YNAILQQETLTYIKNLCDGKN
P.ovale         NKSQNKN.VLYCVYINKYHIIIYHISEPTIIKANHIYQEL.FYEKEKKGIINCHGMNINIRSINIHT..KNSCINNYILNHIKELCDDKK
P.malariae      KKKKENDILLYCAYFNETHIIIYHVGKPKILEPNITYENI.FFEKDKKGIINCYAMDIDIRYVHVYA..TNECVHNYFLEYIKEACQGKK
P.coatneyi      KSKRGND.IIHCAYMNDTHMLIYHVGKPHLLKPNVMYQEI.FFQDSEKGVINCHAMKISLRYVGVHTNGNNNCIQDHLQDHLRNLCNGKK
P.cynomolgi     QNKREGD.LIHCAYMDDKHIVIYHVGKPHLLKPNVVYEEI.FFKDSEKGSISCHAMNISLRYVSINTSGNNNCIQDHLQDYLKNLCNEKK
P.fragile       SSKGKSD.LIHCAYMNDTHIIIYHVGKPQLLKPKVVYKEI.FFQDSEKGVLSCHAMDISLRYVSVHTDSSNNCIQDHFQEHLKQLCNEKK
P.inui          KRKRKSD.LIHCAYMDDKHIVIYHVGKPHVVKPNVVYEEI.FFQEREKGIINCNGMNINLRYIGIHTSDNNSCIQEYFEEHLKKVCNQKR
P.gonderi       KNEKKDS.LTYCAYLNDTHILIYHVGTPQLLKPNVIYEDI.FFKENEKGIITCHAMKINLRYISIHTF.DKKCLQDVFEEHVRAKCNERK
P.berghei       .TEIKND.LLYCAHFNSKELIIYHISEPELLKPDVVYQEIVFLENKKKILINCNTMFVDI..ISINSIPSKYCLNDYLLLYGLKLCTNKT
P.yoelii        .KEIQND.LLYCTHFNNKEMIIYHISEPELLKPDVIYQEIIFLENQKKILINCNTMFIDI..VSINSIPFTYCLNDNLLLYGLKLCANKT
P.chabaudi      .TEIEND.LLYCAHFNSNEMIIYYISEPELLKPDVIYQELLFLENQKKILINCNTMFIEI..VSINSVPSKYCLNDYLLLYALKACTNKT
P.vinckei       .TGIENG.VLYCAHFNSNEMIIYYISEPELLKPDVMYQELLFLENQKKVLINCNTMFIEI..VSINSVPLKYCLNDYLLSYALKSCTNKT

                   220       230       240       250         260       270                           280
P.falciparum    NCKFDFDSIKYEN.KSLTHYLFFINIQYQCISPLNLQE.NEMCDVYNDDTHKATCKYGFNKIE.....................LLKNVC
P.knowlesi      LCEIDFKMVKHEK..CILGSNFLIHVNYECVDRCQQKE.NQTCDIYKGDGRIATCKYGYNALP.....................GISKIC
P.vivax         ACEIDFKNVKKGN.SCSLENNFLIHVNYECVDSCNQKE.NQTCDIYNGEGRMTTCRYGYNMLP.....................RINDFC
P.gallinaceum   NCEINFSEIDKEN.SCLTAHSFYVNIHYECKPRCNIKKEDETCFINDQNSDQTTCKYGYNKLQ.....................LEDEIC
P.relictum      HCEIIFSNVDKSS.KCLTAHSFFVSIYYECKPNCNIKNENEICKATSTETKEVTCKYGYNMLN.....................SDEGTC
P.gaboni        NCKFNFDSIKYEN.KSLTNYLFFINIQYQCKSPLNLRN.NEICDVNNEDINKATCKYGFNRLE.....................LLTNDC
P.reichenowi    NCKFDFDSIKYEN.KSLTYYLFFINIQYQCISPLNLQK.NEMCDVYNEDINKATCKYGFNRIE.....................LLTNAC
P.ovale         YCEIDFDIIKRNE.KCELDEFFVVSINYECEDICNKKE.NEICDLHKGDGKIPTCAYGYNMLP.....................RIKDIC
P.malariae      YCELDFTTIKNGE.NCPMSKNIFITVQYTCVATCNPKL.NEICDIHNSNGLMQTCKYGYNMAI.....................ELSDEC
P.coatneyi      SCEIDFRIVKQGD..CILGSNFLIHVNYECIDSCNPKQ.NQTCDIYNGEGRRATCTYGYNMVP.....................RYKDFC
P.cynomolgi     SCEIDFTKVKSNG..CILGSDFIIHVKYECVDICNQKE.NQTCDIYNGEGRIATCSYGYNMLS.....................SVNNFC
P.fragile       SCEIDFKNIKNED.NCTLGSNFLIHVNYECIDRCNQKE.NETCDIYNGEGRLRTCSYGYNMLP.....................RDKDFC
P.inui          SCEIDFNNVNKGN.NCHLGSNFLIHVNYECEDSCNAKK.NQTCDIYNGEGRVATCSYGYNMLQ.....................GGKETC
P.gonderi       TCEIDFKNIKTGKKKCVLGKDFIVHIKYECIDGCNQEE.NKVCDIYNGEGRLTTCRYGYNMIP.....................IINKFC
P.berghei       YCHIDFSKFNVNS..CPINDSYILHIKYKCKDQPSHILIQNDSANPKIKYSERICKYGYNIIQNSLLNNEIIPNPKSNISIYRKNISNKC
P.yoelii        YCYINFSKFDVKS..CPIYDSYILHIKYRCKEEPNRALIKNDSINSKIKYSERICKYGYNIIQNSLLNKELIYIPKSDIKNYRKFLSNKC
P.chabaudi      YCYINLSKFNLES..CPINDSYSLHIKYKCKESPNLSLSKNDSANSKIKYSERICKYGYNIIQSKLLNKELTYNPKDNINNYRKMISSRC
P.vinckei       YCYINLSKLNLGS..CPINNSYIIQINYRCKEYPSMILSRNESENSKIKYSERICKYGYNIIQNNLLNIELAYNPKGNINSHRKMISNKC
```

```
                                          EGF1                                    EGF2
               290                         300       310       320        330       340       350
P.falciparum   EENYRCTQD....................ICSVNQFCDGENETCTCKTSLL.PSAKNNCEYNDLCTVLNCPENSTCEQIGNGKKAECKCE
P.knowlesi     EKNYKCKSR....................ICSVNEFCDELTESCVCKTSLL.PVEKTQCSYKNVCDAIKCPTNSTCVVDENTKKGECRCE
P.vivax        EKNYTCNSR....................VCSVNQYCDEATESCVCKTSLL.PVEKTHCLYTHVCDAIKCPEDATCVVERNSKKAECRCE
P.gallinaceum  QKNNACLNE....................TCSVNEYCDITERTCKCRNPEL.ESLSNKCESENLCEMLSCPDGATCERASDGKQAECKCE
P.relictum     QKNTACLNE....................ICSVNEYCNTTERSCICRDTIL.DNGNDKCEYDNVCDLLKCPKDASCEYSLDGKKAQCKCS
P.gaboni       EENYRCTQN....................ICSVNQFCDGENETCTCKTSLL.PSAKNNCEYNDLCTVLNCPEHSTCEQIRNGKKAECKCE
P.reichenowi   EENYRCTQD....................ICSVNQFCDGENETCTCKTSLL.PSAKNNCEYNDLCTVLNCPENSTCEQIRSGKKAECKCE
P.ovale        EKNYTCNDK....................ICSLNEYCDNETESCKCKSSLIQPFDSKTCQYDDICKVLNCPENSTCEKIDNGKKAECKCS
P.malariae     ERNYTCGNS...................DICSVNAYCNESSKTCKCKTSLL.PFISNNCVYNNICKVLTCPENSTCEKVDNEEKGECKCQ
P.coatneyi     EKNYTCGNS....................ICSVNEFCDEATKSCVCKTSLV.PVQMTKCSYNNVCDAIKCPANSTCVINGNSKKAECKCN
P.cynomolgi    EKNYTCNGT....................ICSVNQFCDETTKSCLCKTSLL.PLEKNQCFYTNLCDAIKCPEHSTCVVNNNSKKAECRCD
P.fragile      EENYTCNGA....................ICSVNQFCDEETNSCSCKTSDL.PVEGTECLYTDVCKAIECPKDSTCVVTKDSKKAECRCP
P.inui         ERNYTCSGSGGSGSSGKGSNRSNGSSGSGICSVNQFCDEATNSCTCKTSLL.SVEKKECSYTDVCKAMKCPQNSTCALNPKSKKAECKCE
P.gonderi      EKNYTCNNN....................VCSVNEFCSSSSESCVCKTSLVRPEITNNCFYTNVCDVMKCPLKSKCTIYENSKKAECKCE
P.berghei      EKNYTCGNK....................VCSVNQYCDEVSETCKCKKFGI.PINEQTCEPIDVCQLLNCPENSICVQNKQTNKAECKCN
P.yoelii       EKNYSCGNK....................VCSVNQYCDEVTETCRCKKLGI.PINEQTCEPIDVCQLLNCPEDSICVQNKITNKTECKCN
P.chabaudi     EKNYTCGNK....................ICSVNQYCDEATETCKCKNLGI.SINQETCEPIDVCQLLNCPQNSICVKDKTTNKAECKCN
P.vinckei      EKNYTCGNK....................ICSVNQYCDEATETCKCKHLGI.SINQQTCEPIDVCQLLNCPQNSICVKDKITNKAECKCN

                 360       370        380       390       400       410       420       430
P.falciparum   NGKYYHNNKCYTKNDLELAIKIE.PHKKEKFYKNNLYQGKALKPEYIFMQCENGFSIEVINAYVSCYRVSFNLNKL..KYVTESLKKMCD
P.knowlesi     DDKYLYKNKCYSMEELEKLIKTQ.TTPQNKPYKNELFIRSALKPEHIYMHCEDGYFIEVVNATLSCYHVPFRENKI..KYITDRLREICN
P.vivax        EGKYLHKNECYNMAELERLIKAE.TTPHEKQYKKDLFIRSALKPEHIYMHCEEGYSIHVVNATLSCYHISFKENKI..KYITDRLREACN
P.gallinaceum  NDKIFYNNKCYTVEELELAIQLE.PNKKNTIYQDHLHEGSALDSKQIFMKCENDYSIEVVNAYITCYKVQFSNNKM..KYITELLKKECN
P.relictum     DHKIFYNNKCYTPNELELAIQLE.LRKNNTIYKDHLYEGSALQMKHIFLKCEENYSIEVINAYATCYKVQFSDNKM..KYMTEILKKECN
P.gaboni       KGKYYYNNKCYTKNDLELAIKIE.PHKKEKFYKNNLYQGKALKPEHIFMQCENGFSIEVINAYVSCYRVSFNLNKM..KYVTQSLKKMCD
P.reichenowi   NGKYYHNNKCYTKNDLELAIKIE.PHKKEKFYKNNLFQGKALKPEYIFMQCENGFSIEVINAYVSCYRVSFNLNKL..KYVTQSLKKMCD
P.ovale        REKYFYNNKCFNIHELTFEIKME.VTKKEKLYLSNLYEGTSIKHEHIFLECDNNYTIEVVNAYLSCYHISFEKNKM..KYITDYLKKACN
P.malariae     KDKYFYKNRCYNKYDLELAIKMELPIRRRTYYEHTLYDGLSLRPEHIYMQCENGYYIEVVNAYASCYHVSFEKNYM..KYITDYLKRACN
P.coatneyi     DDKYLYKNACYKIEELEKLIEAQ.TTPHHKPYKNALFVRSALRPEHIYMHCEDGYSIEVVNATLSCYHVQFKENKI..KYITDILRGACN
P.cynomolgi    EGKYLHKNRCYDIEEMEKVIKAQ.TTPHHKPYKNELFIRSALKPEYIYMHCEEGYTIEVVNATISCYHITFKENKI..KYITDSLREVCN
P.fragile      DNKYFYKNECYDVGELEELIKKQ.TTTQHKPYKNELFVRSALKPEHIYMECENGYSIEVVNATLSCYHVTFQDNKI..KYITDGLRKACD
P.inui         QGKYFHKNRCYDITEMEKLIKAQ.TTPHDKAYKNELFIRSALKPEHIYMNCEGDYTIEVVNATLSCYNVPFKDNKI..KYITDTLREVCD
P.gonderi      DDKYLYKDKCYDNSELEQVIKME.TTRRDKYYMKDLFTGIALKPEYIFMKCDDGYTIQVVNATSSCYPVIFEKNKI..RYITDRLKEACN
P.berghei      DNKYFYQNKCYYSHEFEDFLKLN.SIEYSQTYIDKLYTGSSLKSEHIFMKCPKDFEIEIVNAYITCYNIPFSLNSVHKKRFTSILKKECN
P.yoelii       DNKYFYQNKCYDSHEFEDFLKLN.HIEYSQTYIDKLYTGSALKPEHIFMECPKDFEIKIINAYASCYNIPFSLNNIHKKRITAILKKECN
P.chabaudi     DNKYYYQNKCYDAHEFEDLLKLN.SIEYNQTYIDKLYIGSSLKSEHIFMKCPADFEIEVVTAYVSCYNIPFSLNSVHKKRPTAILKKECN
P.vinckei      DNKYFYQNKCYDAHEFEDFLKLN.SIEDSQTYIDRLYIGSSLKPEHIFMKCPTDFEIEVVTAYITCYNIPFSLNSIHKKRPTVILKKECN

               440       450        460       470       480                 490                500
P.falciparum   GKTKCTYGNTIDP.IDDLNHHNICNNFNTIFKYDYLCVFNNQN...ITSDKNS........HLHSNIPSLY........NSSILPDINKS
P.knowlesi     GKSRCFFGNSVDKVVPPLDPANTCGTKGNIYQYEYVCVG......................ASTWGTSSSA...............LLQL
P.vivax        GRGRCAFGNSVDQVVPPLDSANMCGTKGTIYQYEYVCVR..........................GGPSP.................PVP
P.gallinaceum  GKMKCVYGNDIDK.VEDTNIFNICDNSNTIFKYQYLCKA...................................................
P.relictum     GKMSCIYGNGVDH..EDTTIYSMCNNSNIVFKYQYLCKA...................................................
P.gaboni       GKNKCAYGNTIDT.IDDLNHDNICSNFNTVFKYDYICEFTNQN...INSDNNS........QLHYNIPSLYNLNMQSSYNSNIRPDMNRS
P.reichenowi   GKTKCAYGNTIDP.IDDLNHHNICNNFNTVFKYDYLCVFNNQN...ITSDMNS........LLHSNIPSLY........NSSILPDINKS
P.ovale        GKRKCAYGNNTDH.IQNTDMTTLCEKGNVIFHYEYLCAKGKEEQSTILYRGNSSLQLF...RRGNYTPSP................IQGE
P.malariae     GKTRCAYGNSIDA.VEHIDIDNMCNEHKVIFHYEYLCAQDDKIGKPLLMLEGSTLPLTSPPQMGIIAPGND........MHNLFVQMQGG
P.coatneyi     GRGRCAFGNSVDE.IPPLDPSNTCGTKGTIYQYEYVCVG......................ATGGGTPSLL...............LPQP
P.cynomolgi    GRNRCAFGNSVDQ.VSPLDKDNICGTKGTIYQYEYMCAG......................ASEVGTPSS................LPRQ
P.fragile      GKARCAFGNSVDQ.VPTLDPGNTCGTKGTIYQYEYVCVG......................ELGGSTSSSP..............SLPLP
P.inui         GRARCAFGNSLKR.VSTLDPLNTCETKGTIYHYEYVCVS......................VPGESSATS................SRAA
P.gonderi      GKVRCAFANRKNP.VLPLDESKMCGEKNVIYEYEYICAQ......................QNNKNIQTNA..............NLSGG
P.berghei      GKNKCVYGSSTDN.TEMLHSPNICTEDFIYFKYNYLCKEKEEFDEEYDEEYDQI.......NENNSEKAKP..............GLKIM
P.yoelii       GKNKCAYGNSIYN.AEILYYPNICTGEFIYFKYNYLCKEKEE.....DDEDDET.......DEHHSEKTKL..............RLENL
P.chabaudi     GKNKCTYGNSANH.VESLHAPNICPNGFIYFKYNYLCKQKDDDDEEEDDEEDG........DSHLSVKTKN..............GLQTF
P.vinckei      GKNKCTYGNSAHH.VESLHTPNVCSHGFIYFKYNYLCKQKEDEDEEEDDEENE........DTHLSVKTKQ..............GLQAF

               510        520       530       540        550       560       570       580       590
P.falciparum   KFHLISRN.SRTNQYPHNNISMLEIQNEISSHNSNQF.STDPHTNSNNINNMNIKKVEIFRSRFSSKLQCQGGKINIDKAILKGGEGCND
P.knowlesi     SVLEPGKPLPSTTRRIMEK.....................................GAIFRSRFNSQIECPGGFITVNRALLKGGDGCED
P.vivax        VMAQLGRD.PPPKSLTKEK.....................................GAIFRSRFHSQIECPGGTITVNKAFLKAGDGCED
P.gallinaceum  .........NNQKDSHKNK...................................EKVEIFRSRYNSKLQCQGGKITLKKALLKTGEGCDD
P.relictum     .........DNQEENHSNK...................................KKVEIFRSRFRSKLQCQGGEITINKALLKTGEGCDD
P.gaboni       KFRLISKN.NRSNQYSRNNLSMIEIQNEISSHNAHQF.GIDPYRNSNNINNMNIKKVEIFRSRFSSKLQCQGGQIIIDKAILKGGEGCND
P.reichenowi   KFRLISRN.SRTNQYPRNNISMLEIQNEISSHNSNQF.STDPNTIGNNINNMNIKKVEIFRSRFSSKLQCQGGQINIDKAILKGGEGCND
P.ovale        AFVQEGIKIKAAHTNTPST........YVTSHLSE...................KKSAVFRSRFESRLHCPGGMITVLKALLKTGDGCDD
P.malariae     SKVHTEGEMKWAAKRTMER........TVSSPVALSVEEINPAQSYNRFK....RKSEIFRSRFSSKIRCDGGTITVTTALLKTGDGCED
P.coatneyi     VLAQRGRG.PSISSYTKEK.....................................GAIFRSRFNSQIECPGGSITVNRALLKAGDGCED
P.cynomolgi    ALIQRGGS.SPQRTHIEGK.....................................GAIFRSRFHSQIECPGGSIRVNKALLKTGDGCED
P.fragile      GLAERTHS.PSRTVHTKDK.......GAVHKNKQEN..................ETGAIFRSRFHSQIECPGGSIKVNKALLKTGDGCQD
P.inui         VWAQLGSD.ASQKIHTKGK.....................................GAIFRSRFNSQLQCPGGSITVNRALLKAGDGCED
P.gonderi      KTITLFKS.EHINNASKEG..................................IGKIAIFRSRFNSKIECNGGSIIVNKALVKTGNGCDD
P.berghei      SKT.IPKNINEHAKIKENK........HATSY........................IKILKNTENSKIECIGGKIQIHSAIIKGGPGCED
P.yoelii       SKNPVSKNINEHTKIKSNQ........NVTSY........................IKILNNTENSKIECLGGKIQIRSAIIKGGPGCED
P.chabaudi     SASPTPKYINEHARRTSNE........SATSY........................IKILKNTENSKIECLGGNIKIRSALIKGGLGCGD
P.vinckei      SPSPIPKYINHHARGASNE........NITSY........................IKILKNTENSKIECPGGKIKIHNAIIKGGSGCGD
```

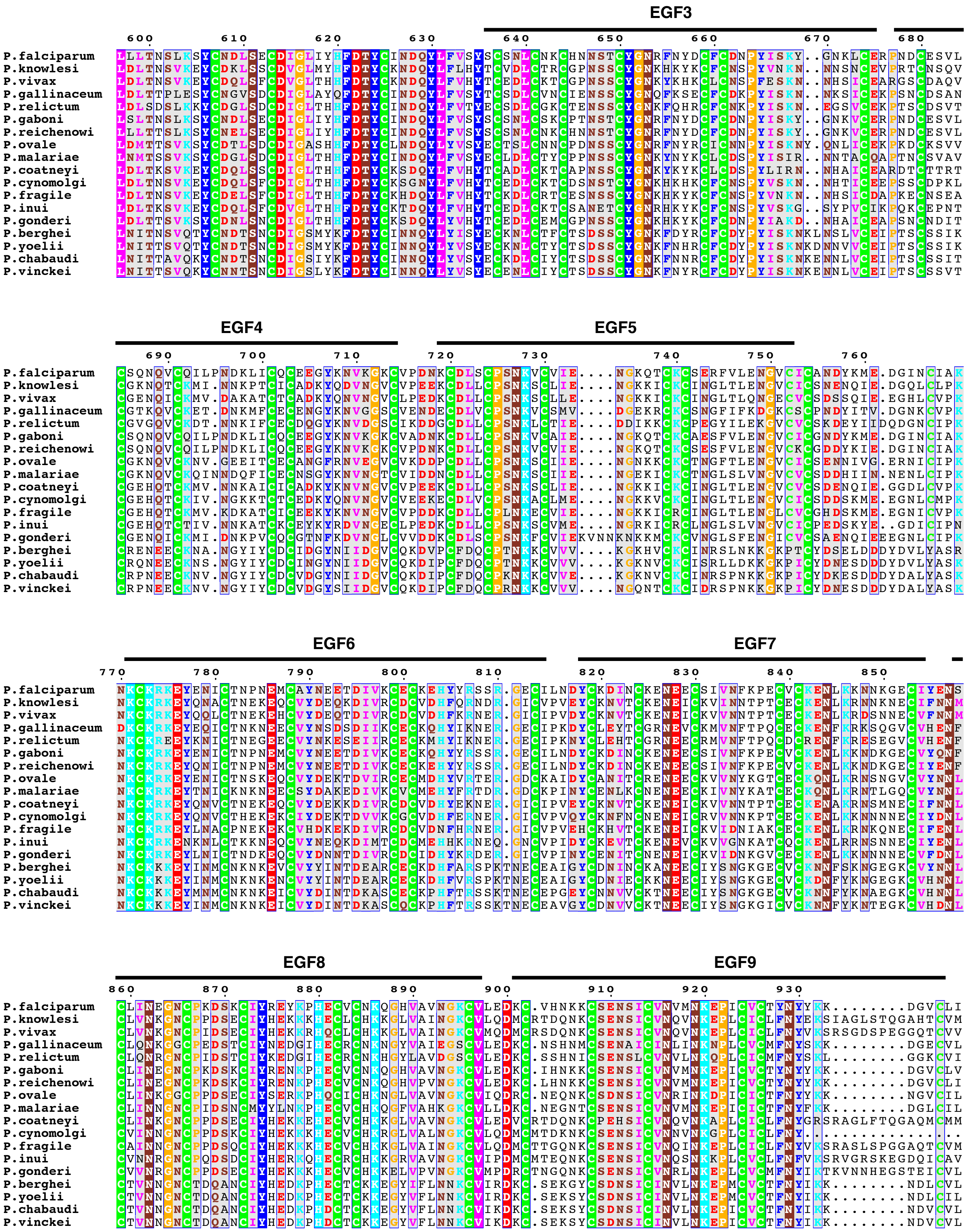
EGF3
600 610 620 630 640 650 660 670 680
P.falciparum LLLTNSLKSYCNDLSECDIGLIYHFDTYCINDQYLFVSYSCSNLCNKCHNNSTCYGNRFNYDCFCDNPYISKY..GNKLCERPNDCESVL
P.knowlesi LDLTNSVKEYCDKLSSCDVGLMYHFDTYCKNDQYLFLHYTCVDLCTRCGPNSSCYGNKHKYKCFCNSPYVNKN..NNSNCEVPRTCNSQV
P.vivax LDLTNSVKEYCDQLSFCDVGLTHHFDTYCKNDQYLFVHYTCEDLCKTCGPNSSCYGNKYKHKCLCNSPFESKN..NHSICEARGSCDAQV
P.gallinaceum LDLTTPLESYCNGVSDCDIGLAYQFDTYCINDQYLFVSYTCSDLCVNCIENSSCYGNQFKSECFCDKPYISKN..NKSICEKPSNCDSAN
P.relictum LDLSDSLKKYCDGLSDCDIGLTHHFDTYCINDQYLFVTYECSDLCGKCTENSSCYGNKFQHRCFCNKPYISKN..EGSVCEKPTSCDSVT
P.gaboni LSLTNSLKSYCNDLSECDIGLIYHFDTYCINDQYLFVSYSCSNLCSKCPTNSTCYGNRFNYDCFCDNPYISKY..GNKVCERPNDCESVL
P.reichenowi LLLTTSLKSYCNELSECDIGLIYHFDTYCINDQYLFVSYSCSNLCNKCHNNSTCYGNRFNYDCFCDNPYISKY..GNKVCERPNDCESVL
P.ovale LDMTTSVKSYCDTLSDCDIGASHHFDTYCLNDQYLYVSYECTSLCNNCPDNSSCYGNRFNYRCICNNPYISKNY.QNLICEKPKDCKSVV
P.malariae LNMTSSVKSYCDGLSDCDIGLTHHFDTYCINDQYLFVSYECLDLCTYCPPNSSCYGNKYNYKCLCDSPYISIR..NNTACQAPTNCSVAV
P.coatneyi LDLTKSVKEYCDQLSSCDIGLTHHFDTYCKSDQYLFVHYTCADLCKTCAPNSSCYGNKYKYKCLCDSPYLIRN..NHAICEARDTCTTRT
P.cynomolgi LDLTNSVKEYCDQLSFCDIGLTHHFDTYCKSGNYLFVHYTCEDLCKTCDSNSTCYGNKHKHKCFCNSPYVSKN..NHTICEEPSSCDPKL
P.fragile LDLTNSVKEYCDELSFCDIGLTHHFDTYCKHDQYLFVHYTCKDLCRTCESNSSCYGNKHKYKCFCNSPYVNKN..NHSICDAPKECNSEA
P.inui LDLTKSVKEYCDQLSFCDVGLTHHFDTYCKTDQYLFVHYTCEDLCKTCSANETCYGNRHKYKCFCNSPYVSKG..SYPVCIKPQKCEPNT
P.gonderi LDLTTSVKSYCDNLSNCDIGLTHHFDTYCKSDQYLYVHYTCEDLCEMCGPNSSCYGNKHKYKCFCDNPYIAKD..NHAICEAPSNCNDIT
P.berghei LNITNSVQTYCNDTSNCDIGSMYKFDTYCINNQYLYISYECKNLCTFCTSDSSCYGNKFDYRCFCDYPYISKNKLNSLVCEIPTSCSSIK
P.yoelii LNITTSVQTYCNDTSNCDIGSMYKFDTYCINNQYLYVSYECKNLCTYCTSDSSCYGNKFNHRCFCDYPYISKNKDNNVVCEIPTSCSSIK
P.chabaudi LNITTAVQKYCNDTSNCDIGSIYKFDTYCINNQYLYVSYECKNLCIYCTSDSSCYGNKFNNRCFCDYPYISKNKENNLVCEIPTSCSSIT
P.vinckei LNITTSVQKYCNNTSNCDIGSLYKFDTYCINNQYLYVSYECENLCIYCTSDSSCYGNKFNYRCFCDYPYISKNKENNLVCEIPTSCSSVT
EGF4
EGF5
690 700 710 720 730 740 750 760
P.falciparum CSQNQVCQILPNDKLICQCEEGYKNVKGKCVPDNKCDLSCPSNKVCVIE....NGKQTCKCSERFVLENGVCICANDYKME.DGINCIAK
P.knowlesi CGKNQTCKMI.NNKPTCICADKYQDVNGVCVPEEKCDLLCPSNKSCLIE....NGKKICKCINGLTLENGVCICSNENQIE.DGQLCLPK
P.vivax CGENQICKMV.DAKATCTCADKYQNVNGVCLPEDKCDLLCPSNKSCLLE....NGKKICKCINGLTLQNGECVCSDSSQIE.EGHLCVPK
P.gallinaceum CGTKQVCKET.DNKMFCECENGYKNVGGSCVENDECDLVCPSNKVCSMV....DGEKRCKCSNGFIFKDGKCSCPNDYITV.DGNKCVPK
P.relictum CGVGQVCKDT.NNKIFCECDQGYKNVDGSCIKDDGCDLLCPSNKLCTIE....DDIKKCKCPEGYILEKGVCVCSKDEYIIDQDGNCIPK
P.gaboni CSQNQVCQILPNDKLICQCEEGYKNVKGKCVADNKCDLLCPSNKVCAIE....NGKQTCKCAESFVLENGVCICGNDYKME.DGINCIAK
P.reichenowi CSQNQVCQILPNDKLICQCEEGYKNVKGKCVPDNKCDLLCPSNKVCVIE....NGKQTCKCSESFVLENGVCKCGDDYKIE.DGINCIAK
P.ovale CGKNQVCKNV.GEEITCECANGFRNVEGVCVKDDPCDLLCPSNKSCIIE....NGNKKCKCTNGFTLENGVCICSENNIVG.ERNICIPK
P.malariae CGKNQVCKQINNDQFICECNSGYKNVNGTCVIDDNCDLLCPSNKSCIIE....NGEKICKCTNGLSLVNGVCVCSDDHIIN.NENLCIPK
P.coatneyi CGEHQTCKMV.NNKAICICADKYKNVNGVCVPEEKCDLLCPSNKSCLIE....NGKQTCKCINGLTLENGVCVCSDENQIE.GGDLCVPK
P.cynomolgi CGEHQTCKIV.NGKKTCTCEDKYQNVNGVCVEEKECDLVCPSNKACLME....NGKKVCKCINGLTLENGVCICSDDSKME.EGNLCMPK
P.fragile CGEHQTCKMV.KDKATCICEEKYKNVNGVCVPDDKCDLLCPLNKECVIE....NGKKICRCINGLTLENGLCVCGHDSKME.EGNICVPK
P.inui CGEHQTCTIV.NNKATCKCEYKYRDVNGECLPEDKCDLLCPSNKSCVME....NGRKICRCLNGLSLVNGVCICPEDSKYE..GDICIPN
P.gonderi CGENQICKMI.DNKPVCQCGTNFKDVNGLCVVDDKCDLSCPSNKSCFVIEKVNNKNKKMCKCVNGLSFENGICVCSAENQIEEEGNLCIPK
P.berghei CRENEECKNA.NGYIYCDCIDGYNIIDGVCQKDVPCFDQCPTNKKCVVV....KGKHVCKCINRSLNKKGKPTCYDSELDDDYDVLYASR
P.yoelii CRQNEECKNS.NGYIYCDCINGYNIIDGVCQKDIPCFDQCPTNKKCVVV....KGKNVCKCISRLLDKKGKPICYDNESDDDYDVLYASK
P.chabaudi CRPNEECKNV.NGYIYCDCVDGYNIIDGVCQKDIPCFDQCPKNKKCVVE....KGKNVCKCINRSPNKKGKPICYDKESDDDYDALYASK
P.vinckei CRPNEECKNV.NGYIYCDCVDGYSIIDGVCQKDIPCFDQCPRNKKCVVV....NGQNTCKCIDRSPNKKGKPICYDNESDDDYDALYASK
EGF6
EGF7
770 780 790 800 810 820 830 840 850
P.falciparum NKCKRKEYENICTNPNEMCAYNEETDIVKCECKEHYYRSSR.GECILNDYCKDINCKENEECSIVNFKPECVCKENLKKNNKGECIYENS
P.knowlesi NKCKRKEYQNACTNEKEQCVYDEQKDIVRCDCVDHFQRNDR.GICVPVEYCKNVTCKENEICKVINNTPTCECKENLKRNNKNECIFNNM
P.vivax NKCKRKEYQQLCTNEKEHCVYDEQTDIVRCDCVDHFKRNER.GICIPVDYCKNVTCKENEICKVVNNTPTCECKENLKRDSNNECVFNNM
P.gallinaceum DKCKRKEYEQICTNKNEECVYNSDSDIIKCECKQHYIKNER.GECIPKDYCKDINCGRNEECKMVNFTPQCECKENFKRKSQGVCVHENF
P.relictum NKCKREEYKNICTNEGEECVYNKESEIIRCECKMHYIKNER.GECIPKNYCLEHTCGRNEECRMVNFTPQCDCRENFKRESEGVCVHENF
P.gaboni NKCKRKEYENICTNPNEMCVYNEETDIVKCECKQHYYRSSR.GECILNDYCKDINCKENEECSIVNFKPECVCKENLKKNDKGECVYQNF
P.reichenowi NKCKRKEYQNICTNPNEMCVYNEETDIVKCECKEHYYRSSR.GECILNDYCKDINCKENEECSIVNFKPECVCKENLKKNDKGECIYENF
P.ovale NKCKRKEYENICTNSKEQCVYDEKTDVIRCECMDHYVRTER.GDCKAIDYCANITCRENEECKVVNYKGTCECKQNLKRNSNGVCVYNNL
P.malariae NKCKRKEYTNICKNKNEECSYDAKEDIVKCVCMEHYFRTDR.GDCKPINYCENLKCNENEECKIVNYKATCECKQNLKRNTLGQCVYNNL
P.coatneyi NKCKRKEYQNVCTNEKEQCVYDEKKDIVRCDCVDHYEKNER.GICIPVEYCKNVTCKENEICKVVNNTPTCECKENAKRNSMNECIFNNL
P.cynomolgi NKCKRKEYQNVCTHEKEKCIYDEKTDVVKCGCVDHFERNER.GICVPVQYCKNFNCNENEICRVVNNKPTCECKENLKKNNNNECIYDNL
P.fragile NKCKRKEYLNACPNEKEKCVHDEKEDIVRCDCVDNFHRNER.GICVPVEHCKHVTCKENEICKVIDNIAKCECKENLKRNKQNECIFENL
P.inui NKCKRKENKNLCTKKNEQCVYNEQKDIMTCDCMEHHKRNEQ.GNCVPIDYCKEVTCKENEVCKVVGNKATCQCKENLRRNSNNECIYENL
P.gonderi NKCKRKEYLNICTNDKEQCVYDNNTDIVRCDCIDHYKRDER.GICVPINYCENITCNENEICKVIDNKGVCECKENLKKNNNNECVFDNL
P.berghei NKCKKKEYINMCNKNKEVCVYYINTDEARCECKDHFARSPKTNECEAIGYCDNINCKANEECIYSNGKGECVCKNNFSKNGEGKCVYNNL
P.yoelii NKCKKKEYINMCNKNKENCVYYINTDEARCECKDHFVRSPKTNECEAIGYCDNIECKKNEECIYSNGKGECVCKDNFYKNGEGKCVHNNL
P.chabaudi NKCKKKEYMNMCNKNKEICVYDINTDKASCECKPHFTRSSKTNECEPGEYCNNVVCKTNEECIYSNGKGECVCKNNFYKNAEGKCVHNNL
P.vinckei NKCKKKEYINMCNKNKEICVYDINTDKASCQCKPHFTRSSKTNECEAVGYCDNVVCKTNEECIYSNGKGICVCKNNFYKNTEGKCVHDNL
EGF8
EGF9
860 870 880 890 900 910 920 930
P.falciparum CLINEGNCPKDSKCIYREYKPHECVCNKQGHVAVNGKCVLEDKC.VHNKKCSENSICVNVMNKEPICVCTYNYYKK........DGVCLI
P.knowlesi CLVNKGNCPPDSECIYHEKKKHECLCHKKGLVAINGKCVLQDMCRTDQNKCSENSICVNQVNKEPLCICLFNYEKSIAGLSTQGAHTCVM
P.vivax CLVNKGNCPIDSECIYHEKKRHQCLCHKKGLVAINGKCVMQDMCRSDQNKCSENSICVNQVNKEPLCICLFNYVKSRSGDSPEGGQTCVV
P.gallinaceum CLQNKGGCPEDSTCIYNEDGIHECRCNKNGYVAIEGSCVLEDKC.NSHNMCSENAICINLINKNPLCVCMFNYSKK........DGECVL
P.relictum CLQNRGNCPIDSTCIYKEDGIHECRCNKHGYLAVDGSCVLEDKC.SSHNICSENSLCVNVLNKQPLCICMFNYSKL........GGKCVI
P.gaboni CLINEGNCPKDSKCIYRENKPHECVCNKQGHVAVNGKCVLEDKC.IHNKKCSENSICVNVMNKEPICVCTYNYYKK........DGVCLV
P.reichenowi CLINEGNCPKDSKCIYRENKPHECVCNKQGHVPVNGKCVLEDKC.LHNKKCSENSICVNVMNKEPICVCTYNYYKK........DGVCLI
P.ovale CLINKGGCPPDSECIYSERKPHQCICHKNGLVAVNGKCVIQDRC.NEQNKCSDNSICVNRINKDPICICTFNYYKK........NGVCIL
P.malariae CLINNGNCPIDSNCMYYLNKPHECVCHKQGFVAHKGKCVLLDKC.NEGNTCSENSICVNVMNKEPICICTFNYFKK........DGLCIL
P.coatneyi CLINKGNCPPDSECIYHEKKEHECVCHKKGLVAVNGKCVLEDVCRTDQNKCPEHSICVNQVNKAPLCICLFNYGRSRAGLFTQGAQMCMM
P.cynomolgi CVINNGNCPPDSKCIYHEKKKHECVCHKRGLVALNGKCVLQDMCMTDKNKCSENSICVNNVNKGPLCICLFNY................
P.fragile CAINNGNCPPDSQCIYHEKKKHECVCHKKGLVAINGKCVLQDMCTTGQNKCSENSICVNQINKEPLCICLFNYVKSRASLSPGGAQTCVM
P.inui CVNNRGNCPQDSDCIYHERKQHECRCHKKGRVAVNGKCVIPDMCMTEQNKCSENSICVNQSNKKPLCVCLFNFVKSRVGRSKEGDQICAV
P.gonderi CVVNRGNCPPDSECIYHEKKKHECVCHKKELVPVNGKCVMPDRCTNGQNKCSENSICVNRLNKEPLCVCMFNYIKTKVNNHEGSTEICVL
P.berghei CTVNNGNCTDQANCIYHEDKPHECTCKKEGYIFLNNKCVIRDKC.SEKGYCSDNSICINVLNKEPMCVCTFNYIKK........NDLCVL
P.yoelii CTVNNGNCTDQANCIYHEDKPHECTCKKEGYVFLNNKCVIRDKC.SEKSYCSDNSICINVLNKEPMCVCTFNYIKK........NDLCIL
P.chabaudi CTVNNGNCTDQANCIYHEDKPHDCTCKKEGYVFLNNKCVIKDKC.SEKSYCSDNSICINVLNKEPICVCTFNYIKK........NDVCVL
P.vinckei CTVNNGNCTDQANCIYHEDKPHDCTCKKEGYVFLNNKCVIKDKC.SEKSYCSDNSICINVLNKEPICVCTFNYIKK........NDVCVL

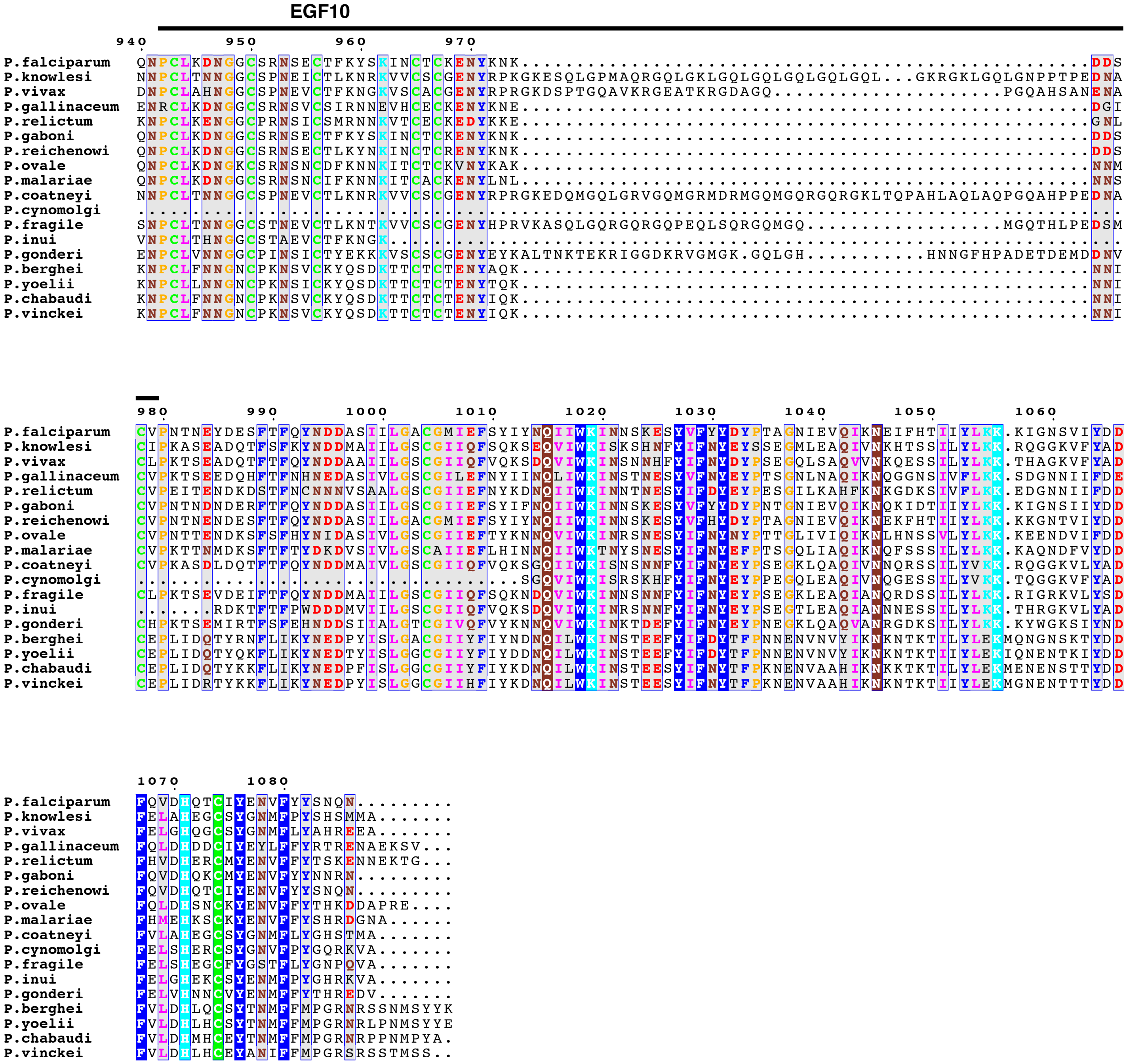
EGF10
940 950 960 970
P.falciparum QNPCLKDNGGCSRNSECTFKYSKINCTCKENYKNK..DDS
P.knowlesi NNPCLTNNGGCSPNEICTLKNRKVVCSCGENYRPKGKESQLGPMAQRGQLGKLGQLGQLGQLGQLGQL...GKRGKLGQLGNPPTPEDNA
P.vivax DNPCLAHNGGCSPNEVCTFKNGKVSCACGENYRPRGKDSPTGQAVKRGEATKRGDAGQ.....................PGQAHSANENA
P.gallinaceum ENRCLKDNGGCSRNSVCSIRNNEVHCECKENYKNE..DGI
P.relictum KNPCLKENGGCPRNSICSMRNNKVTCECKEDYKKE..GNL
P.gaboni QNPCLKDNGGCSRNSECTFKYSKINCTCKENYKNK..DDS
P.reichenowi QNPCLKDNGGCSRNSECTLKYNKINCTCRENYKNK..DDS
P.ovale QNPCLKDNGKCSRNSNCDFKNNKITCTCKVNYKAK..NNM
P.malariae QNPCLKDNGGCSRNSNCIFKNNKITCACKENYLNL..NNS
P.coatneyi NNPCLTNNGGCSPNEVCTLKNRKVVCSCGENYRPRGKEDQMGQLGRVGQMGRMDRMGQMGQRGQRGKLTQPAHLAQLAQPGQAHPPEDNA
P.cynomolgi ....
P.fragile SNPCLTNNGGCSTNEVCTLKNTKVVCSCGENYHPRVKASQLGQRGQRGQPEQLSQRGQMGQ..................MGQTHLPEDSM
P.inui VNPCLTHNGGCSTAEVCTFKNGK...
P.gonderi ENPCLVNNGGCPINSICTYEKKKVSCSCGENYEYKALTNKTEKRIGGDKRVGMGK.GQLGH...........HNNGFHPADETDEMDDNV
P.berghei KNPCLFNNGNCPKNSVCKYQSDKTTCTCTENYAQK..NNI
P.yoelii KNPCLLNNGNCPKNSICKYQSDKTTCTCTENYTQK..NNI
P.chabaudi KNPCLFNNGNCPKNSVCKYQSDKTTCTCTENYIQK..NNI
P.vinckei KNPCLFNNGNCPKNSVCKYQSDKTTCTCTENYIQK..NNI
980 990 1000 1010 1020 1030 1040 1050 1060
P.falciparum CVPNTNEYDESFTFQYNDDASIILGACGMIEFSYIYNQIIWKINNSKESYVFYYDYPTAGNIEVQIKNEIFHTIIYLKK.KIGNSVIYDD
P.knowlesi CIPKASEADQTFSFKYNDDMAIILGSCGIIQFSQKSEQVIWKISKSHNFYIFNYEYSSEGMLEAQIVNKHTSSILYLKK.RQGGKVFYAD
P.vivax CLPKTSEADQTFTFQYNDDAAIILGSCGIIQFVQKSDQVIWKINSNNHFYIFNYDYPSEGQLSAQVVNKQESSILYLKK.THAGKVFYAD
P.gallinaceum CVPKTSEEDQHFTFNHNEDASIVLGSCGILEFNYIINQLIWKINSTNESYVFNYEYPTSGNLNAQIKNQGGNSIVFLKK.SDGNNIIFDE
P.relictum CVPEITENDKDSTFNCNNNVSAALGSCGIIEFNYKDNQIIWKINNTNESYIFDYEYPESGILKAHFKNKGDKSIVFLKK.EDGNNIIFDD
P.gaboni CVPNTNDNDERFTFQYNDDASIILGSCGIIEFSYIFNQIIWKINNSKESYVFYYDYPNTGNIEVQIKNQKIDTIIYLKK.KIGNSIIYDD
P.reichenowi CVPNTNENDESFTFQYNDDASIILGACGMIEFSYIYNQIIWKINNSKESYVFHYDYPTAGNIEVQIKNEKFHTIIYLKK.KKGNTVIYDD
P.ovale CVPNTTENDKSFSFHYNIDASIVLGSCGIIEFTYKNNQVIWKINRSNESYIFNYNYPTTGLIVIQIKNLHNSSVLYLKK.KEENDVIFDD
P.malariae CVPKTTNMDKSFTFTYDKDVSIVLGSCAIIEFLHINNQIIWKTNYSNESYIFNYEFPTSGQLIAQIKNQFSSSILYLKK.KAQNDFVYDD
P.coatneyi CVPKASDLDQTFTFQYNDDMAIVLGSCGIIQFVQKSGQIIWKISNSNNFYIFNYEYPSEGKLQAQIVNNQRSSILYVKK.RQGGKVLYAD
P.cynomolgi ..................................SGQVIWKISRSKHFYIFNYEYPPEGQLEAQIVNQGESSILYVKK.TQGGKVFYAD
P.fragile CLPKTSEVDEIFTFQYNDDMAIILGSCGIIQFSQKNDQVIWKINNSNNFYIFNYEYPSEGKLEAQIANQRDSSILYLKK.RIGRKVLYSD
P.inui .......RDKTFTFPWDDDMVIILGSCGIIQFVQKSDQVIWKINRSNNFYIFNYEYPSEGTLEAQIANNNESSILYLKK.THRGKVLYAD
P.gonderi CHPKTSEMIRTFSFEHNDDSIIALGTCGIVQFVYKNNQVIWKINKTDEFYIFNYEYPNEGKLQAQVANRGDKSILYLKK.KYWGKSIYND
P.berghei CEPLIDQTYRNFLIKYNEDPYISLGACGIIYFIYNDNQILWKINSTEEFYIFDYTFPNNENVNVYIKNKNTKTILYLEKMQNGNSKTYDD
P.yoelii CEPLIDQTYQKFLIKYNEDTYISLGGCGIIYFIYDDNQILWKINSTEEFYIFDYTFPNNENVNVYIKNKNTKTIIYLEKIQNENTKIYDD
P.chabaudi CEPLIDQTYKKFLIKYNEDPFISLGGCGIIYFIYKDNQILWKINSTEESYIFNYTFPKNENVAAHIKNKKTKTILYLEKMENENSTTYDD
P.vinckei CEPLIDRTYKKFLIKYNEDPYISLGGCGIIHFIYKDNQILWKINSTEESYIFNYTFPKNENVAAHIKNKNTKTIIYLEKMGNENTTTYDD
1070 1080
P.falciparum FQVDHQTCIYENVFYYSNQN.........
P.knowlesi FELAHEGCSYGNMFPYSHSMMA.......
P.vivax FELGHQGCSYGNMFLYAHREEA.......
P.gallinaceum FQLDHDDCIYEYLFFYRTRENAEKSV...
P.relictum FHVDHERCMYENVFFYTSKENNEKTG...
P.gaboni FQVDHQKCMYENVFYYNNRN.........
P.reichenowi FQVDHQTCIYENVFYYSNQN.........
P.ovale FQLDHSNCKYENVFFYTHKDDAPRE....
P.malariae FHMEHKSCKYENVFFYSHRDGNA......
P.coatneyi FVLAHEGCSYGNMFLYGHSTMA.......
P.cynomolgi FELSHERCSYGNVFPYGQRKVA.......
P.fragile FELSHEGCFYGSTFLYGNPQVA.......
P.inui FELGHEKCSYENMFPYGHRKVA.......
P.gonderi FELVHNNCVYENMFFYTHREDV.......
P.berghei FVLDHLQCSYTNMFFMPGRNRSSNMSYYK
P.yoelii FVLDHLHCSYTNMFFMPGRNRLPNMSYYE
P.chabaudi FVLDHMHCEYTNMFFMPGRNRPPNMPYA.
P.vinckei FVLDHLHCEYANIFFMPGRSRSSTMSS..
