# Supplementary material for: Divergent roles for the RH5 complex components, CyRPA and RIPR in human-infective malaria parasites
Source: PLoS Pathog. 2019 Jun 11;15(6):e1007809. doi: 10.1371/journal.ppat.1007809 (PMC6588255; doi:10.1371/journal.ppat.1007809)
Supplement: S5 Fig — We used anti-HA antibodies (green) to localize HA-PTRAMP, HA-CSS and CyRPA-HA in the respective transgenic parasite lines (indicated on the left of each image row) and co-localized this signal with antibodies to the micronemal marker AMA1 (red, top panels) but not with antibodies to the rhoptry bulb marker RhopH2 (red, bottom panels). Nuclei are stained with DAPI (blue). Scale bar = 2 μm. (PDF) [file ppat.1007809.s005.pdf]

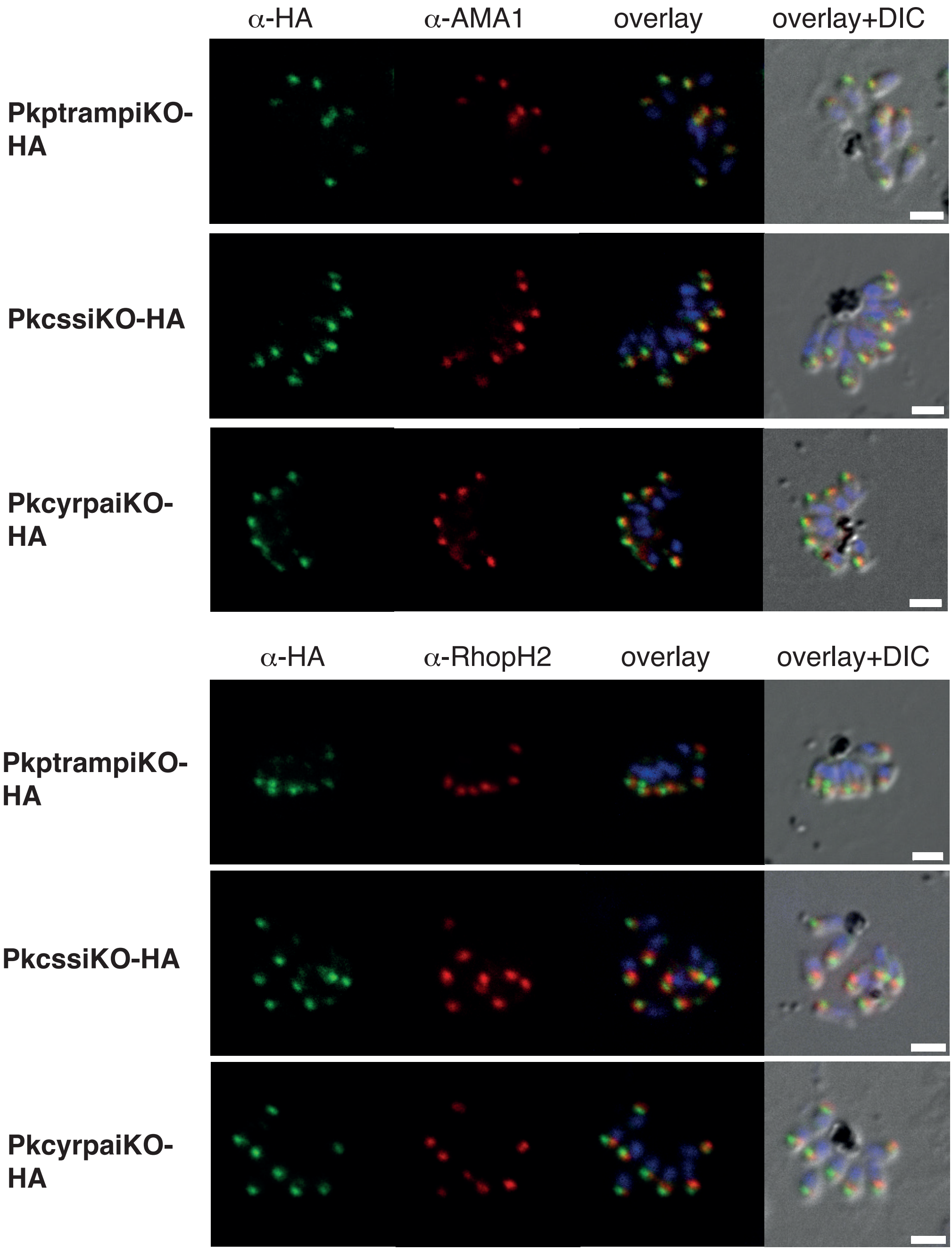
α-HA
α-AMA1
overlay
overlay+DIC
PkptrampiKO-HA
PkcssiKO-HA
PkcyrpaiKO-HA
α-HA
α-RhopH2
overlay
overlay+DIC
PkptrampiKO-HA
PkcssiKO-HA
PkcyrpaiKO-HA
