# Supplementary material for: Divergent roles for the RH5 complex components, CyRPA and RIPR in human-infective malaria parasites
Source: PLoS Pathog. 2019 Jun 11;15(6):e1007809. doi: 10.1371/journal.ppat.1007809 (PMC6588255; doi:10.1371/journal.ppat.1007809)
Supplement: S1 Table — The treatment conditions used for each isolate, which are reported in Fig 1E, are indicated by tick marks. (PDF) [file ppat.1007809.s009.pdf]

S. ID	Basigin_mAb (10 μg/ml)	Basigin_pAb (10 μg /ml)	Mouse_IgG (10 μg /ml)	Goat_IgG (10 μg/ml)	Control well parasitemia (%)
Pv004		√	√		6.3
Pv023	√	√	√	√	2.18
Pv024	√	√	√	√	2.0
Pv025	√	√	√	√	7.75
Pv019	√	√			2.48
Pv027		√			1.21
Pv032	√	√	√	√	1.97
Pv020	√	√	√	√	3.45
Pv033	√	√			2.6
Pv041	√	√			1.38
