# Supplementary material for: Divergent roles for the RH5 complex components, CyRPA and RIPR in human-infective malaria parasites
Source: PLoS Pathog. 2019 Jun 11;15(6):e1007809. doi: 10.1371/journal.ppat.1007809 (PMC6588255; doi:10.1371/journal.ppat.1007809)
Supplement: S2 Table — Restriction enzyme sites within the oligonucleotide DNA sequence are listed on the right. (PDF) [file ppat.1007809.s010.pdf]

Primer name	Abbreviated name	DNA sequence	restriction enzyme sites
pHH4riprfor		TTTAATCCCGGGGTGCAGACAAATACCAAGATGTG	XmaI
pHH4riprrev		TTTAATCCTAGGAGCCATCATCGAGTGACTGTAGG	AvrII
HA rev	3	CCTTTACCGCGGTCAAGCGTAATCTGGAACGTCGTAAGGGTAGCCCATGG	SacII
PKriprextF1	1	CCAATTGTGAAGTACCACGAACATGC	
PKriprutrRev1	2	CAATTAATGTGACAATTTTCGCTCTCTTC	
PKRIPRkoextFor	a	GCATACAAGACTACCTCCATGATCATC	
PKRIPRintRev	b	CGTCAACAACGCAAGTGGAGTTTGT	
PKRIPRintFor	c	GAGAACACGAAGAAGGGAGAGTGC	
PKRIPRkoextRev	d	CACCCACACACACATACTCGTACTG	
PKRIPRkorecRev	e	GTATTTTCATCCACTACACATGTACTATTG	
PKRIPRkorecFor	f	GTGGATGAAAATACAAAAAAGGGGGAATG	
HArevXmaSTOP		TATTCTCCCGGGCTAAGCGTAATCTGGAACGTCGTAAGGG	XmaI
Harev(Xma)	g	TATTCTCCCGGGAGCGTAATCTGGAACGTCGTAAGGG	XmaI
HAfor(Xma)	h	AACAAACCCGGGTACCCTTACGATGTTCCTGACTATGC	XmaI
PKCyRPAextFor	i	CGCGACTGACTGACCTCTAAAGAG	
PKCyRPAintRev	j	CAACGATCACAAGCGACTCCTTC	
PKCyRPAintFor	k	GTGATCGTTGGAGTAGCACCACG	
PKCyRPAextRev	l	TTGAGTTCGTCGGGTTTAGGATACAC	
PKCyRPArecRev	m	GTTGTAGATGACCATCTGTCGCATGC	
PKCyRPArecFor	n	GCATGCGACAGATGGTCATCTACAAC	
PDC596for		ATTGCAACGCAAGTGGAGTTTGT	
pDC596rev		AAACACAAACTCCACTTGCGTTG	
pDC645for		ATTGCACGAAGAAGGGAGAGTGC	
pDC645rev		AAACGCACTCTCCCTTCTTCGTG	
pDC2915for		ATTGGATCGTTGGAGTAGCACCA	
pDC2915rev		AAACTGGTGCTACTCCAACGATC	
pDC2919for		ATTGAGGAGTCGCTTGTGATCGT	
pDC2929rev		AAACACGATCACAAGCGACTCCT	
Pkhsp86promforNcoSpeI		GCTATACCATGGTTTTATACTAGTAAAACACTTCACCCACGTCGTG	NcoI; SpeI
Pkhsp86promRevAvrII		TTGGTTCCTAGGTTTGGCTTAGCAGGGTTGGCAAAATG	AvrII
Pkef1aFor(SpeI)		ACCTGCACTAGTTAAGTAACCCTTGCATATGCCC	SpeI
Pkef1aRev(NcoI)		TTTCTGCCATGGTTTCGAATAAAATTAAATTGAAAAAAAGG	NcoI
pDC45for		ATTGCAAATGGTGCATAGCAGCA	
pDC45rev		AAACTGCTGCTATGCACCATTTG	
pDC982for		ATTGAGTTGTACGAAAATGTGGC	
pDC982rev		AAACGCCACATTTTCGTACAACT	
PkptrampExtFor	4	ATAGCAATCGCTCACCATTAGGCACATAAG	
PkptrampExtRev	5	ATTACAGCCTGGTCGGGAAGGGATTTTG	
PkptrampIntRev	6	ATTGCTACCAACGGAGAAATTAAAGTCCCC	

PkptrampRecodRev	7	ACTGTACATTCTGGCTCCAGGGTTACTATC	
PkptrampIntFor	8	GGAGAGCTTTCGTAAAGGACAGACTTACG	
PkptrampRecodFor	9	CCTATCATTCTGATTATCTGCGTCATGGGC	
pDC89for		ATTGGTGGGTGCTAAAAGGGCGG	
pDC89rev		AAACCCGCCCTTTTAGCACCCAC	
pDC1081for		ATTGTATTGAGCCCTGTCTGCTT	
pDC1081rev		AAACAAGCAGACAGGGCTCAATA	
PkCSSextForB	10	AATGAGCAAATTTGTGGCTAGCCAATTCCTG	
PkCSSextRevB	11	ATGTTCACAATTTTGCGTAGGCGGCAGC	
PkCSSintRevB	12	CATCGTTTCTATCGATGGACACGCCTTTTC	
PkCSSintForB	13	CCAGATGAACATGGAATACACGCTTGTAAC	
PkCSSrecodRevB	14	GCGGCCCTCTTCGCCCTCTGCCTT	
PkCSSrecodForB	15	CTGGACATTGTGACCAACCAACTGCTTCC	
pDC591for		ATTGGAAGACAAGGGAACAACTT	
pDC591rev		AAACAAGTTGTTCCCTTGTCTTC	
pDC1229for		ATTGTTCTGAACACCGTGTCCAG	
pDC1229rev		AAACCTGGACACGGTGTTCAGAA	
PkCyRPAextForB	16	GGAAGGACGGAGATCCATGTCATCGTGC	
PkCyRPAextRevB	17	CCTGTCATATCAGTTGTGATTGGAAAGTCC	
PkCyRPAintRevB	18	GGACGAAGGTAGAAGTATGGAACTCCTTC	
PkCyRPArecodRevB	19	GGCACACCCTCCTGGAATTGATCGTAATAG	
PkCyRPAintForB	20	GAGGACAAGTCTCTTCAAAACATTACTAAGC	
PkCyRPArecodForB	21	CAGATCGCGGCGGAAAGTACATGAAATGC	
PKhsp86promFBglSac		AATGAAGATCTAAATATCCGCGGAAAACACTTCACCCACGTCGTG	BglII; SacII
Pkhsp86promrevXho		AATAGTCTCGAGTTTGGCTTAGCAGGGTTGGCAAAATG	XhoI
PK47HR1for2BglII		AATAGTAGATCTCACATCTAACGGATTACATAGCAATG	BglII
PK47HR1rev2SacII		AATACTCCGCGGCATTTTCCTTGGCATCCAAAATTCTATC	SacII
PK47HR2forEcoRV		AATAGAGATATCGGAGATTATCAACATGGATTTCC	EcoRV
PK47HR2rev2Short		AATACAGATATCGTTCCATAATTCCTGTCTTTCCAC	EcV
pDC946for		ATTGAATCAAGAACCTAAAATAG	
pDC946rev		AAACCTATTTTAGGTTCTTGATT	
